# Manual annotation and analysis of the defensin gene cluster in the C57BL/6J mouse reference genome

**DOI:** 10.1186/1471-2164-10-606

**Published:** 2009-12-15

**Authors:** Clara Amid, Linda M Rehaume, Kelly L Brown, James GR Gilbert, Gordon Dougan, Robert EW Hancock, Jennifer L Harrow

**Affiliations:** 1 Wellcome Trust Sanger Institute, Wellcome Trust Genome Campus, Hinxton, Cambridgeshire CB10 1SA, UK; 2 University of British Columbia, Centre for Microbial Disease & Immunity Research, 2259 Lower Mall, Vancouver, BC, V6T 1Z4, Canada; 3 Department of Rheumatology and Inflammation Research, Göteborg University, Guldhedsgatan 10, S-413 46 Göteborg, Sweden

## Abstract

**Background:**

Host defense peptides are a critical component of the innate immune system. Human alpha- and beta-defensin genes are subject to copy number variation (CNV) and historically the organization of mouse alpha-defensin genes has been poorly defined. Here we present the first full manual genomic annotation of the mouse defensin region on Chromosome 8 of the reference strain C57BL/6J, and the analysis of the orthologous regions of the human and rat genomes. Problems were identified with the reference assemblies of all three genomes. Defensins have been studied for over two decades and their naming has become a critical issue due to incorrect identification of defensin genes derived from different mouse strains and the duplicated nature of this region.

**Results:**

The defensin gene cluster region on mouse Chromosome 8 A2 contains 98 gene loci: 53 are likely active defensin genes and 22 defensin pseudogenes. Several TATA box motifs were found for human and mouse defensin genes that likely impact gene expression. Three novel defensin genes belonging to the Cryptdin Related Sequences (CRS) family were identified. All additional mouse defensin loci on Chromosomes 1, 2 and 14 were annotated and unusual splice variants identified. Comparison of the mouse alpha-defensins in the three main mouse reference gene sets Ensembl, Mouse Genome Informatics (MGI), and NCBI RefSeq reveals significant inconsistencies in annotation and nomenclature. We are collaborating with the Mouse Genome Nomenclature Committee (MGNC) to establish a standardized naming scheme for alpha-defensins.

**Conclusions:**

Prior to this analysis, there was no reliable reference gene set available for the mouse strain C57BL/6J defensin genes, demonstrating that manual intervention is still critical for the annotation of complex gene families and heavily duplicated regions. Accurate gene annotation is facilitated by the annotation of pseudogenes and regulatory elements. Manually curated gene models will be incorporated into the Ensembl and Consensus Coding Sequence (CCDS) reference sets. Elucidation of the genomic structure of this complex gene cluster on the mouse reference sequence, and adoption of a clear and unambiguous naming scheme, will provide a valuable tool to support studies on the evolution, regulatory mechanisms and biological functions of defensins *in vivo*.

## Background

Defensins are the largest family of cationic host defense peptides in humans, and possess immunomodulatory and direct antimicrobial activities [1]. In humans, alpha-defensins are most abundant in neutrophils and Paneth cells [2]. There are rare human disorders (Chediak Higashi Syndrome and Specific Granule Deficiency) associated with decreased or absent neutrophil alpha-defensins, however other neutrophil granule components are also deficient which makes it difficult to assign these disorders to defensins themselves [3]. Loss or down regulation of defensin genes is related to certain types of human cancer [4-6]. Since murine neutrophils lack defensins [2,7], Paneth cells provide an alternative to study alpha-defensins in discrete compartments in a model organism, the mouse, which has the largest known repertoire of defensin-encoding sequences. The discovery of a mouse Paneth cell defensin peptide, termed cryptdin (Defcr) due to its expression in the Crypts of Lieberkühn [8], was the first report of defensin expression in a non-myeloid cell lineage [9,10]; *Defcr *was subsequently mapped to mouse Chromosome 8 [11,12] and since has been discovered to be part of a larger gene family including additional alpha-defensin genes as well as cryptdin-related sequences (CRS), also known as Defcr-rs (Defcr-related sequence). This is due to their sequence similarity and genetic linkage to *Defcr *[9-13]. Additional Defcr/Defcr-rs loci have been discovered in different mouse strains, some of which may be polymorphic and/or involved in copy number variation [11,14-17]. The confusion around gene names, variable copy numbers and polymorphisms has made the study of mouse defensins quite complex.

Defensin peptides are characterized by six canonical cysteine residues at defined positions in the amino acid sequence. The different spacing pattern between these cysteines and the arrangement of the three disulphide bonds that link them allow their further classification into three subfamilies: alpha-, beta- and theta-defensins [18-20]. Beta-defensins have a broad tissue expression pattern and have been found across most vertebrates and some invertebrate species, whilst alpha-defensins are specific to certain mammals and are mainly produced by leukocytes of myeloid origin and Paneth cells of the small intestine [18-20]. Theta-defensins are believed to be derived by cyclization of alpha-defensins and seem to be restricted to the leukocytes of Old World monkeys [21].

Defensin genes have a characteristic two-exon structure, and this is true for most mouse alpha-defensin genes. However there are exceptions within the alpha-defensin family, some of which have three exons. Members of the beta-defensin family can have between two to four exons, for example fish and birds have three-exon beta-defensins [22]. However the differences between alpha- and beta-defensins are most likely a consequence of gene duplication and subsequent divergence selected during evolution [23]. Extensive analysis has provided insight into the evolution of mammalian beta-defensins [23-25]. Alpha-defensins are thought to have arisen by repeated gene duplication of beta-defensins and positive diversifying selection [23,26]. Therefore mouse Paneth cell alpha-defensins have most likely "lost" one of these exons during evolution and gene/chromosome duplication events have led to their two exon structure. The high similarity of mouse alpha-defensin genes and subsequent repetitive nature of their chromosomal position lends support to this model. As a rapidly evolving gene family, defensins provide a useful system through which to study mammalian evolution.

The nomenclature of mouse alpha-defensins is complicated due to the duplicated nature of the genes. Problems are encountered when mining genome databases Ensembl, MGI and NCBI since there are multiple references to individual gene names, making it difficult to identify the actual annotated gene on the reference sequence. The manual annotation presented here of the defensin region on mouse Chromosome 8 addresses the nomenclature issues for the alpha-defensins and collaboration with the Mouse Genome Nomenclature Committee (MGNC) is helping to resolve these issues.

A collaborative effort was established between the Centre for Microbial Disease & Immunity Research at the University of British Columbia (Vancouver, BC/Canada) and the Wellcome Trust Sanger Institute (Hinxton, Cambridge/UK) to investigate the genomic structure of alpha-defensins and their functionality.

## Results and Discussion

### Genomic overview of the annotated region on mouse Chromosome 8

To fully characterize the defensin genes, we initially annotated a genomic region on mouse Chromosome 8 consisting of 18 finished BAC clones spanning 2.4 Mb of the NCBI Build 36 assembly (and subsequently the Build 37) reference sequence. To date this is the only known region associated with alpha-defensins and was therefore chosen as the initial starting point for defensin annotation in mouse. Known MGI nomenclature was used to name genes only if there was a 100% cDNA match. Otherwise we referred to an interim Vega database identifier such as OTTMUSG00000018259. The Vega database identifier is stable, versioned and will remain unchanged after further naming or assembly updates. MGNC has started assigning symbols to most defensin pseudogenes identified here and also implementing some of our suggestions concerning nomenclature (Additional file 1: Supplemental Tables S1&S2).

**Figure 1:**
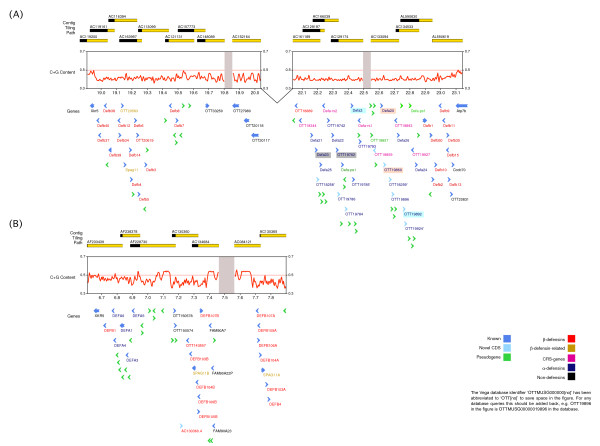
**Overview of the defensin gene cluster region in mouse (top) and human (bottom)**. A clone tiling path is shown for the corresponding regions in mouse (top) and human (bottom). Clones are displayed in yellow but regions overlapping with adjacent clones are shown in black. Genes are indicated by arrows. Genes in shadowed boxes are duplicated and the color indicates the pairs; A -'- highlights all potential *Defcr5 *genes (see color legend for more details). The mouse assembly is based on NCBIM37, in which three gaps currently exist; two gaps are indicated by grey bars and the biggest gap between the two clusters is joined by a 'V'.

This region contains three gaps of various sizes ranging from 50 Kb to 2 Mb where additional/missing defensin genes could be located (Figure 1A, map of the whole region including gaps). Two beta-defensin gene clusters flank the region containing the alpha-defensin genes. In total, annotation of this genomic region revealed the existence of 54 and 44 loci respectively in the alpha-defensin and beta-defensin gene clusters (Additional file 1: Supplemental Tables S1&S2). The entire defensin gene cluster is flanked by *Xkr5 *(X Kell blood group precursor-related family, member 5) centromeric to *Ccdc70 *(coiled-coil domain containing 70), *Atp7b *(ATPase, Cu++ transporting, beta polypeptide) and *Alg11 *(asparagine-linked glycosylation 11 homolog (yeast, alpha-1,2-mannosyltransferase)).

### Beta-Defensin cluster

The mouse beta-defensin cluster contains 27 beta-defensin genes, including sperm-associated antigen 11 (*Spag11*) and OTTMUSG00000020593 (*Spag11c/h*). *Spag11 *genes encode beta-defensin-like peptides and have shown tissue- and species-specific alternative splicing in primate species [27]. Furthermore, three beta-defensin pseudogenes and nine other pseudogenes as well as a gene coding for a novel protein with tubby-like domains have been annotated (Figure 1A). This manual annotation confirms the mouse beta-defensin repertoire reported in the most recent studies on mammalian beta-defensins [24,28]. In the human genome most beta-defensin genes have been recently duplicated but in the mouse genome our manual annotation did not reveal any 100% identical beta-defensin genes. This analysis is however limited by the current mouse genome assembly in that we might not have been able to see the most recent duplications. A very recent publication indicates that a duplicated region is missing in the current assembly [29]. Finishing of this region might still reveal duplicated beta-defensin genes similar to those in the alpha-defensin gene set (see below).

### Alpha-Defensin cluster

**Figure 2:**
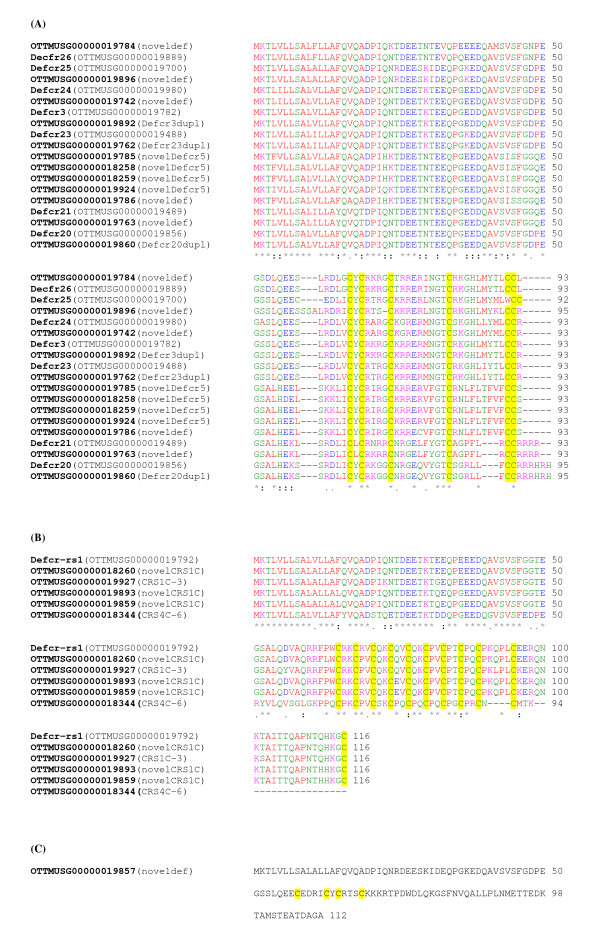
**Multiple protein alignment of defensin peptides**. Most defensin peptides contain six canonical cysteine residues (A); Members of the CRS1C family contain eleven cysteines in a different spacing between each other; CRS4C-6 belongs to the CRS4C family but consists of ten instead of the usual nine cysteines for this group (B). A novel sequence (OTTMUSG00000019857) has been annotated within the defensin gene cluster region which lacks all the canonical cysteines in any known number and spacing. Four cysteine residues can be found here but they don't align with any of the known cysteines in other peptides (C). All cysteine residues are highlighted in yellow. Genes identified for the first time in this study are tagged as noveldef.

**Figure 3:**
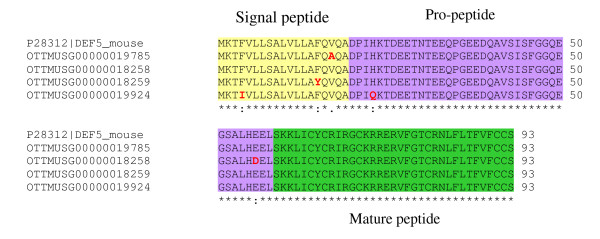
**The polymorphic *Defcr5 *locus**. A protein alignment between all potential *Defcr5 *copies and P28312.2. Variation in amino acids is highlighted in red.

Twenty six apparently intact defensin-related cryptdin genes and 22 related pseudogenes were observed within the mouse alpha-defensin cluster (Figure 1 and 2A). Furthermore six MYM-Type zinc finger protein pseudogenes as well as three ribosomal protein pseudogenes are also located in this region. Within the alpha-defensin gene cluster there is a region containing several genes very similar to Defcr5 but no identical match of the Swiss-Prot entry P28312.2 for Defcr5, which is derived from the genomic sequence of the 129 mouse strain. Two of these loci, OTTMUSG00000019785 and OTTMUSG00000018259 show only one amino acid difference in their signal peptides compared to the Defcr5 Swiss-Prot entry P28312.2 (Figure 3). Locus OTTMUSG00000018258 shows one amino acid difference in its pro-segment to P28312.2 and locus OTTMUSG00000019924 differs in one amino acid in the signal peptide and one in the pro-segment compared to P28312.2. These genes all have identical mature peptides compared to the P28312.2 Defcr5 sequence and have therefore been tagged as novel protein similar to defensin related cryptdin 5. Questions arise as to whether a common sequence for the mature peptide qualifies these genes to be named the same as a published sequence, whether they have the same functionality and how differences in the signal- and/or pro-segment might affect their expression. Consequently, these *Defcr5 *loci might be the result of chromosomal duplications or involved in copy number variation similar to a number of defensin genes where we observed 100% identity throughout the entire sequence (see below). Locus OTTMUSG00000019786 also has a best match to Defcr5 but there are three amino acid differences, one in the signal peptide, one in the pro-segment and another one in the mature peptide compared to P28312.2. Therefore, this locus has been annotated as a novel defensin related cryptdin without commenting on any similarity to Defcr5, since there are clear precedents for applying different names to defensins with small sequence changes. In some cases 100% identical copies of a gene were identified. One example of this is represented by two copies for *Defcr23*. To clarify this situation we have tagged one copy as *Defcr23 *and the other one as 'novel defensin related cryptdin identical to *Defcr23*'. Two other alpha-defensin genes, *Defcr3 *and *Defcr20*, have duplications in the mouse genome. Genes with duplicated copies are ideal candidates for copy number variation.

### Cryptdin-related sequences

Within the alpha-defensin gene cluster (Figure 1A) we identified genes that show similarity to the prosegment of the alpha-defensins but a different number and spacing pattern of cysteines compared to any other known antimicrobial peptides [9,10,30]. Such genes are usually referred to as cryptdin-related sequences (CRS). We have annotated six genes belonging to two groups of cryptdin-related sequences, CRS1C and CRS4C (Figure 2B) [13,30]. Nine cysteine residues characterize CRS peptides of the CRS4C class found in the gastrointestinal mucosa [31]; *CRS4C-6*, annotated here OTTMUSG00000018344 belongs to the subfamily CRS4C; however, CRS4C-6, which harbors ten cysteine residues has not been included in prior studies [9,10,30]. Another cryptdin-related sequence CRS1C-3 OTTMUSG00000019927, which is located within the alpha cluster, is characterized by the presence of 11 cysteines. An alpha-defensin-related sequence *Defcr-rs*, also known as *CRS1C-2 *and OTTMUSG00000018260 which shows three amino acid differences, one in the signal sequence and two in the mature peptide, compared to Defcr-rs1 OTTMUSG00000019792, was also found in the alpha gene cluster.

Two identical genes have been assigned as coding for novel CRS1C peptides, OTTMUSG00000019859 and OTTMUSG00000019893. All CRS1C peptides known to date encode 116 amino acid proteins in contrast to alpha-defensins that encode proteins with 93-95 amino acids. A study on CRS4C peptides has shown that they form covalent homo- and heterodimers, *in vitro *and *in vivo*, and are potent at killing commensal and pathogenic bacteria, *in vitro*[31]. Whether Defcr-rs1 (CRS1C-2) with its 11 cysteines has similar capabilities is unknown but it shares all nine cysteines with members of the CRS4C family and all ten with CRS4C-6.

We have tried to determine computationally whether any members of the CRS family can be found in any other species, rat in particular. Results can be viewed in Additional file 1: Supplemental Tables S3&S4, Additional file 2: Supplemental Information S1 and Additional file 3: Supplemental Figures S1&S2.

### Identification of new splice variants within the mouse defensin genes

**Figure 4:**
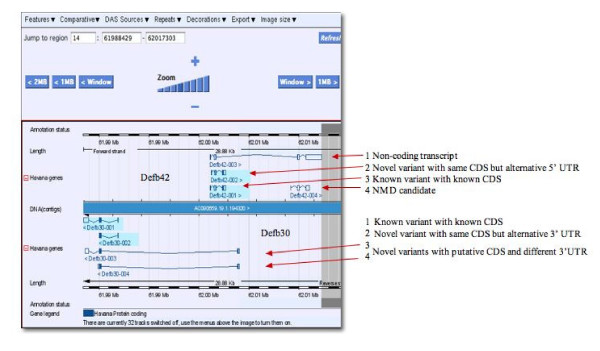
**Novel coding and non-coding variants**. Vega presenting the region for Defb30 and Defb42, where three new variants per locus were annotated. *Defb30*: Variants 1 is a known variant with known CDS, variant 2 is a novel variant with the same CDS as variant 1 but has an alternative 3' UTR, variant 3 and 4 are novel variants with putative CDS and different 3'UTR. *Defb42*: Variant 1 represents a non-coding transcript, variant 2 is a novel variant with the same CDS as the known transcript (3) but with an alternative 5' UTR, variant 3 is a known variant with known CDS and variant 4 is a NMD candidate.

To complete the defensin gene set in mouse all other loci on Chromosomes 1, 2 and 14 were also annotated. The beta-defensin cluster on Chromosome 2 consisting of 11 gene loci is the largest among them. Interestingly, novel splice variants were annotated for *Defb30 *and *Defb42 *on Chromosome 14, which is in contrast to the family members on Chromosome 8. *Defb30 *has four different splice variants, one of which was previously known; three variants have been tagged as "putative coding" as they have a different first exon compared to the known variant. Two pairs of variants share the same 5' exon but differ in the 3' exons. In each pair, one variant consists of three exons and the other one of two (Figure 4). For *Defb42 *two coding and two non-coding variants have been identified and annotated. One of the transcripts that seems to lack coding properties has been tagged as a transcript likely to be subject to nonsense-mediated mRNA decay (NMD). All four *Defb42 *variants have differentially spliced 5' first exon and only one has previously been known in other gene sets. Tissue-specific and species-specific alternative splicing has been previously shown for primate SPAG11 [27]. The beta-defensin *Defb42 *has been discovered and characterized in mice and its expression has been shown to be epididymis-specific [32]. Looking at the origin of the manually annotated spice variants for *Defb42 *it is noticeable that all cDNA clones representing the main coding variant are derived from the adult male reproductive tract, specifically the epididymis. However, there is one coding cDNA with an alternative 5' UTR exon compared to the main variant that has been derived from the spleen of a four week old male mouse. The potential NMD splice variant is a two cells egg cDNA and another overlapping non-coding transcript is based on an 11 days embryo whole body cDNA. This observation suggests that alternative splicing for *Defb42 *is likely to be also development stage specific. An unusual feature was observed for *Defb17 *and *Defb41 *on Chromosome 1. These genes share the same start and first exon but differ in their second exon, which is crucial since it encodes the mature peptide. According to our general annotation guidelines these two genes would normally be merged and the two transcripts would represent splice variants of the same gene since they share the first coding exon. Differential splicing seems to be a rare event for defensin genes; however, the observed examples here indicate the potential functional differences for the affected transcripts.

### Genomic structures of annotated defensins on Chromosome 8

#### A) TATA Boxes

**Figure 5:**
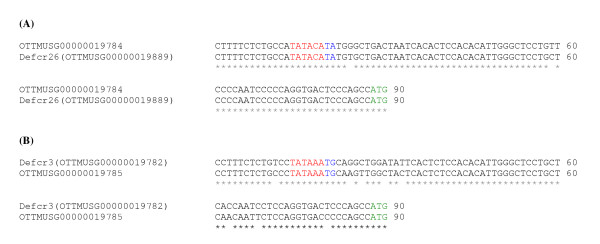
**Potential promotor region for some defensin genes**. (A) For two genes OTTMUSG00000019784 and *Defcr26 *a weak TATA box motif could be identified. (B) A strong TATA box motif was found for 27 defensin genes, here an example is shown for *Defcr3 *and OTTMUSG00000019785, a novel defensin gene. TATA box motifs are shown in red/blue and start codons in green.

Annotation of TATA boxes has been based on motifs verified experimentally and published previously for five defensin genes in mouse [14,15] and two defensin genes in human [33]. We suggest the position of TATA box motifs for several more defensin genes (26 in total) by manual annotation of this gene cluster in mouse (Figure 5 and Additional file 1: Supplemental Table S1) with duplicated defensin genes having the same TATA box sequence. However, for some loci no TATA box could be defined based on the known consensus. For a novel alpha-defensin gene, OTTMUSG00000019784, and for *Defcr26 *a TATA box with a weaker consensus was identified (Figure 5) which may affect the expression of these genes. An experimental verification would be necessary to find out whether this motif is active and if so to what extent. It is known that TATA box containing genes are significantly more likely to change in expression and are biased towards spontaneous mutations [34]. Three genes in the beta-defensin region (*Defb12*, *Defb51 *and *Defb33*) and a defensin-related gene (*Spag11c/h*) show a three-exon structure and the gene *Defb52 *consists of four exons in contrast to all the other defensin genes with two exons. A TATA box could only be identified for one of them, *Defb12*, with a consensus identical to the main TATA box motif in this region. In the human cluster, the annotation of TATA box motifs revealed the existence of one motif (TTAAATA) that has not been identified in mouse.

Several TATA box-less genes (*Defcr25*, OTTMUSG00000019857 and OTTMUSG00000019896) and also two pseudogenes (OTTMUSG00000019793 and OTTMUSG00000019923) have an identical 5' UTR/promoter region to that of previously reported 'Crypi' [14,15], which is presumed to be non-functional because of a pre-mature stop codon. The question that arises is whether these loci represent new pseudogenes or whether their expression is regulated by an alternative promoter. The gene OTTMUSG00000019857 has a divergent C-terminus/mature peptide compared to all other defensin genes (Figure 2C). It has a coding potential for 112 amino acids but three of the consensus cysteine residues are missing. These data suggest that this gene is possibly pseudogenic in the reference genome, C57BL/6J.

#### B) Pseudogenes

A total of 22 defensin pseudogenes were annotated in the major mouse defensin gene cluster region on mouse Chromosome 8 (Additional File 1: Supplemental Table S2), while ten pseudogenes were annotated in the corresponding human defensin cluster. It has been shown that ~10% of the pseudogenes annotated in the Encyclopedia of DNA Elements (ENCODE) project are from genes involved in the immune response [35], thus the high frequency of defensins pseudogenes (>25%) is notable.

We divide pseudogenes into two categories, processed and unprocessed, with two subcategories each, transcribed or untranscribed. A locus is annotated as pseudogene when clear homology is shown to proteins but the coding sequence is disrupted, resulting in frameshifts or in-frame stop codons. A locus can also be tagged as a pseudogene when one or more parent genes that show spliced gene structure can be found elsewhere in the genome while the pseudogene locus looks like a single exon encoding for the corresponding protein.

Pseudogenes of the defensin gene cluster are unprocessed as they have likely evolved through duplication of functional genes and have accumulated mutations over time and become non-functional. Of the 22 pseudogenes annotated in this region five are only partial and one, *Defa-ps1 *has been tagged as transcribed_unprocessed pseudogene. Here, protein homologies point to this locus being a pseudogene, but overlapping locus-specific transcription evidence (cDNAs) indicates expression. There is recent evidence that regulatory interdependency exists between transcribed pseudogenes and their parent gene. For example a targeted knockdown of the transcribed ABC transported pseudogene *ABCC6P1 *results in a significant reduction of the parent gene *ABCC6 *expression levels [36]. As an ongoing collaboration between our group and the MGNC, a list of annotated pseudogenes has been sent to MGNC and symbols have been already assigned to some of them (Additional file 1: Supplemental Table S2).

We have also looked at the 5' upstream genomic sequences of defensin related pseudogenes to look for TATA box-like motifs. There are two defensin pseudogenes where a strong TATA box motif (TATAAA TG) could be found and four pseudogenes with a weak TATA box motif (TATACA TA/G), but for the majority nothing similar could be identified. Looking at the transcribed pseudogene, *Defa-ps1*, the homology breaks 48 bp upstream of the start codon and no TATA box-like motif could be identified. Generally, the TATA box-lacking defensin pseudogenes have 5' sequences similar to the potential promoter regions of TATA box-lacking active defensin genes.

### Comparative Analysis of Gene Sets

To illustrate the difficulties created in naming the defensins, by comparison to the assembled data from databases and the literature, we have cross-referenced four major gene sets and assembled gene symbols and gene IDs for the mouse alpha-defensin genes annotated herein (Additional file 1: Supplemental Table S5). These include our manually curated data set from Vega v.30, automatic gene annotation from Ensembl v.49, cDNA evidence from NCBI RefSeq, and a merged gene set encompassing UCSC, RefSeq and Ensembl from MGI 4.01 [37]. The gene *Defcr25 *has a cross-reference to gene *Defcr2 *in NCBI's Entrez Gene indicating that this gene is also known as *Defcr2*. However, the protein sequences for Defcr25 (MGI:3630385; Swissprot:Q5G864.1) and Defcr2 (MGI:94882; Swissprot:P28309.2) are different and are derived from different mouse strains. Therefore, we have annotated the locus as *Defcr25*, since the sequence in the reference mouse genome is identical to this gene. Another example of ambiguities between databases are *Defcr16 *and *Defcr17*, which have been associated by MGI with OTTMUSG00000019742 and OTTMUSG00000019892 respectively; in case of *Defcr16 *evidence for its expression is derived from C3H/HeJ strain only and the association of *Defcr17 *with the Vega model is incorrect as OTTMUSG00000019892 is a duplicate of *Defcr3*. Additional examples are listed in the Additional file 1: Supplemental Table S5.

This analysis has highlighted the necessity in accounting for strain differences when deriving gene annotation based on cDNAs aligned to the C57BL/6 reference genome. The differences become very obvious when looking at the alpha-defensin region in MGI Genome Browser (Additional file 3: Supplemental Figures S3&S4). The number of alpha-defensin genes here is higher than the number annotated by our group but looking at the origin of many of the genes reveals that they are derived from strains distinct from the reference genome. An example is the Crp4 peptide, which was first isolated from Outbred Swiss mice [38] and corresponds to Defcr4 in MGI; this gene has been annotated on the 129X1/SvJ strain but has not been annotated on the reference strain (Additional file 1: Supplemental Table S6). The reference strain contains two presumed Crp4 peptide variants termed Crp4-B6a and Crp4-B6b because they are all missing three codons between the forth and fifth cysteine residues [39]. MGNC has named these variants Defcr20 and Defcr21, respectively; however the relationship of these peptides between the two mouse strains is not obvious.

Data displayed in MGI's GBrowse is a combination of down loaded information from the UCSC's Genome browser [40] and that generated at MGI. MGI and UCSC do not filter any strain-specific data and mapping of defensin genes has not been carried out stringently with the aim of displaying a comprehensive set of all existing genes. This is a valuable resource, however, as a result several genes which share the same nucleotide sequence coding for the signal peptide and the pro-segment have been mapped together. However, as the region encoding the mature peptide shows some differences these genes cannot be considered the same.

To determine the ease of cross-species comparison, genomic alignments and putative orthologues were searched for in both human and rat genomes compared to mouse alpha-defensins. There are only six defensin genes with defined orthologues between human and mouse found on the Mouse Chromosome 8 Linkage Map [41]. We appreciate that for the mouse alpha-defensin family, orthology is especially hard to predict because of the high intraspecies similarity for these genes. The same human and rat genes align with most of the mouse genes and/or are predicted to be orthologues for the mouse peptides. In particular, DEFA7P is predicted to be the human orthologue for the majority of the mouse cryptdins by Ensembl but this gene lacks a start codon [23] and has therefore been designated a pseudogene by manual annotation. These alignments and orthologues have been predicted by Ensembl and may be an artefact of their naming scheme. We also examined the human and rat genome assemblies and compared a region of conserved synteny relative to the mouse genome (Additional file 2: Supplemental Information S2).

Since defensin genes are involved in copy number variation as well as they reveal polymorphisms between strains and a one to one orthology between different species is hard to predict, it is crucial to work out a standardized system that can be followed by all genome browsers to indicate these important differences. As a first step towards this solution we propose a reorganization of the nomenclature of the mouse alpha-defensins.

### Time to update the defensin naming scheme?

Consistency and standardization in naming genes is often an issue between research groups, journals and genome browsers. Additional file 1: Supplemental Table S7 gives an overview of the defensin naming in various species, which highlights these discrepancies. This is very obvious in the case of the defensin genes in mouse and is most likely due to a combination of factors. Defensins were first discovered as peptides or from cDNA. With the completion of large-scale genome sequencing projects, it has become possible to mine these genomes for defensin genes by scanning translated genomic sequence for the six conserved cysteine residues. Some defensins have only been identified at the genomic level without subsequent peptide or RNA expression data. Searching the literature gives the impression that the naming of cryptdins is orderly and systematic however searching major databases for the corresponding information is difficult. The problem arises because most of the experimental data comes from mouse strains different to the C57BL/6J reference genome. We have found evidence to confirm that there are strain and CNV differences within the defensin gene family and therefore annotating/mapping genes and peptides discovered in non-reference strains remains a challenge.

A recent report has reviewed the nomenclature for chicken defensins, also known as 'gallinacins', and proposed their standardized renaming as a result of confusion in the literature from the employment of multiple naming schemes by different groups [42]. Currently, the naming for beta-defensins in avians is very heterogeneous (e.g. 'ostricacins' for ostrich beta-defensins) and a system has been proposed that involves renaming genes as part of an adaptation to the new system for avian beta-defensins in which the term "avian beta-defensin", abbreviated to AvBD, has been suggested [42].

Here we propose a similar reconsideration of mouse, rat and human defensins with the aim of a standardized naming scheme agreed on by the corresponding nomenclature committees, MGNC/RGNC/HGNC, which we hope will be adopted by the major genome centres, journals and genome browsers so that results from various research studies can be compared easily and efficiently. We also propose that the naming scheme should be applicable to all organisms. For human, mouse and many other species, the abbreviation DEFB/Defb and DEFA/Defa has been used to tag beta- and alpha-defensins, respectively. Both HGNC and MGNC groups have approved the DEFB/Defb and DEFA/Defa naming schemes for human and mouse [43]. We propose the continuation of the latter system as otherwise a taxonomy-based naming would become complicated; therefore we have sent our suggestions for a clearer and unified naming scheme to MGNC. The change of the Defcr root to Defa has already been implemented for mouse alpha-defensins. We propose that the naming scheme should also take into consideration relationships between peptides in different strains of mice (e.g. as shown for Crp4).

Since alpha-defensins are being found in species outside of the *Euarchontoglires *branch of mammals [44], it is crucial to establish a standardized nomenclature system. Also the existence of CNV in defensin genes, and of genes with highly related sequences, adds to the complexity of developing a consistent and meaningful naming scheme.

## Conclusions

The investigation of mouse alpha-defensins is difficult due to the duplicated nature of the genes and the resulting gaps within the reference genome assembly of the alpha-defensin region of mouse Chromosome 8. Knocking out multiple endogenous defensins, including alpha-defensins, in mice may provide a direct indication of their function and also address the issue of redundancy; however difficulties in characterizing mouse alpha-defensins has hindered this research. Designing unique primers for individual knockout of alpha-defensin genes is impossible and the exact complement of the alpha-defensin genes within the mouse reference genome (C57/BL6 strain) is still unknown. The recent formation of the Genome Reference Consortium [45] should contribute to gap closure in important regions such as the alpha-defensin on Chromosome 8 and aid identification of the complete defensin repertoire for the reference mouse genome. Detection of copy number variation and structural variation in mouse, similar to what has been observed in human (Additional file 2: Supplemental Information S3) has proven to be difficult because of the limited sequence information on various mouse strains and as well the presence of the 2 Mb gap in Chromosome 8. This gap has the potential to be collapsed due to the highly repetitive nature of the genes in this region but also if there is a larger scale duplication. Current mouse tile-path arrays do not have the capability to resolve individual gene copy numbers and their design is limited by the current mouse genome assembly. Genes that we have annotated as 100% identical are obvious candidates for copy number variants but sequence information from additional mouse strains is needed for this verification. Between the existing alpha-defensins we have observed apparent polymorphisms in different mouse strains. A project undertaken at the Sanger Institute aims to sequence the genomes of 17 common mouse strains, but it is reasonable to assume that there will be difficulties in assembling Chromosome 8 proximal to regions containing alpha-defensin genes. However comparisons between sequence available for these strains will start to define the copy number and polymorphic variation of mouse alpha-defensins.

The manual annotation of the defensin gene cluster enabled not only the identification of many pseudogenes previously unknown in this region, but also the identification of novel defensin genes and defensin-related sequences belonging to the CRS family with a different cysteine arrangement. The experimental validation to confirm if these proteins are functional is yet to be done.

At present only a small fraction of the annotated genes have peptide products that have been purified. The small size, redundancy and variable expression levels of these peptides may be the reasons for the difficulty in isolating the peptides. Transcriptional profiling will enable the identification of expressed genes, however sophisticated methods will be necessary to distinguish between and quantify these highly similar transcripts. Additionally it can be determined whether expression correlates with the promoter region for each gene, i.e. the presence of a TATA box, CpG island or novel motif. This will allow for the separation of the roles of each unique defensin in normal immune function as well as during infection or inflammation.

Evolution, duplication and allelic variation of defensin genes are currently under investigation [46-48]. Since many diseases/disorders appear to be modulated by copy number variation, the review and standardization of CNV nomenclature is critical to future studies.

## Methods

### Analysis and Annotation pipeline

Prior to the process of manual annotation an automated analysis for similarity searches and *ab initio *predictions is run in an extended Ensembl analysis pipeline system [49]. All search results are stored in an Ensembl MySQL database. Following genomic sequence masking of interspersed repeats and tandem repeats by RepeatMasker and Tandem repeats finder [50], a search with wuBLASTN against the nucleotide databases starts. Significant hits are then re-aligned to the unmasked genomic sequence using est2genome [51]. The Uniprot protein database is then searched with wuBLASTX. In order to provide prediction of protein domains Genewise [52] is used to align hidden Markov models for Pfam protein domains against the genomic sequence. Finally, a number of different *ab initio *algorithms are used: Genescan [53] for genes, tRNAscan [54] to find tRNA genes and Eponine TSS [55], to predict transcription start sites.

After completion of the automated analysis, manual annotation starts using a Perl/Tk based graphical interface, called 'otterlace', developed in-house to edit annotation data stored in a separate MySQL database system [56]. It provides different tools for changing exon coordinates, adding gene names and remarks, assigning genes to different categories or adding genomic features such as poly(A) sites and signals. The annotation of gene objects requires a visual representation of the genomic region and features like CpG islands, repeats and poly(A) sites, gene predictions, evidence which support the annotation of gene structures (ESTs/cDNAs/proteins) and all transcript variants created by annotators. This representation is provided by a graphical user interface called ZMap which was written in C to give the high performance required to display large numbers of features (Storey, personal communication). An alignment viewer called 'Blixem' [57] allows gapped alignments of nucleotide and protein blast hits to be compared with the genomic sequence. Furthermore, a 'Dotplot' tool called 'Dotter' [57] is used to show pair-wise alignments of unmasked sequences, revealing the location of exons that are occasionally missed by the automated blast searches because of their small size and/or match to repeat-masked sequence.

All annotation is publicly available in the Vertebrate Genome Annotation (VEGA) browser. Definitions of Vega gene and transcript types can also be found on the website http://vega.sanger.ac.uk/index.html. Since the first annotation of the region was performed the assembly has changed slightly and some of the gene names have been changed. Figure 1 represents the most current situation and Vega will be updated accordingly.

### Mouse genomic assembly

This manual annotation of the defensin gene cluster region was based on NCBI Build 36. However, at the time of the writing the new build NCBIM37 has been released and the two assemblies show several differences. The most crucial one is that a new clone AC161189 has been added to the new assembly which overlaps partially with and has replaced clone AC140205. Although most genes initially annotated in AC140205 are present in AC161189, there are seven loci missing. One of these codes for beta-defensin 33 (*Defb33*) and the remaining ones are different pseudogenes. In order to preserve this data we propose that the clone AC140205 should be trimmed from the point where it is unique and be returned to the new assembly. The Genome Reference Consortium [45], which is a collaborative effort between NCBI, the Sanger Institute, EMBL-EBI and the Genome Center at Washington University, aim to close remaining gaps in the human and mouse genomes and remove discrepancies in clones observed by research groups. The issue described here has been submitted to the Consortium.

## Abbreviations

EST: expressed sequence tag; cDNA: complementary DNA; MGNC: Mouse Genomic Nomenclature Committee; RGNC: Rat Genomic Nomenclature Committee; HGNC: Human Genomic Nomenclature Committee.

## Authors' contributions

CA carried out the genomic annotation. CA and LMR performed the genomic analysis and co-wrote the manuscript. KLB has set up the cooperation between the Sanger Institute, UK and the Centre for Microbial Disease & Immunity Research, Canada. REWH supports the PhD studies of LMR. GD supported LMR during her research exchange at the Sanger Institute. JLH provided support throughout the project. JLH, REWH and GD were involved in critical discussions and manuscript revisions. All authors read and approved the final manuscript.

## Supplementary Material

Additional file 1 Supplemental Tables Click here for file

Additional file 2 Supplemental Information Click here for file

Additional file 3 Supplemental Figures Click here for file

## References

[B1] Ganz T Selsted M Lehrer R Defensins Eur J Haematol 1990 44 1 8 2407547 10.1111/j.1600-0609.1990.tb00339.x

[B2] Mestas J Hughes CCW Of Mice and Not Men: Differences between Mouse and Human Immunology J Immunol 2004 172 2731 2738 14978070 10.4049/jimmunol.172.5.2731

[B3] Ganz T Metcalf J Gallin J Boxer L Lehrer R Microbicidal/cytotoxic proteins of neutrophils are deficient in two disorders: Chediak-Higashi syndrome and "specific" granule deficiency Journal of Clinical Investigation 1988 82 552 556 10.1172/JCI113631 2841356 PMC303547

[B4] Schullerus D von Knobloch R Chudek J Herbers J Kovacs G Microsatellite analysis reveals deletion of a large region at chromosome 8p in conventional renal cell carcinoma Int J Cancer 1999 80 22 24 9935224 10.1002/(SICI)1097-0215(19990105)80:1<22::AID-IJC5>3.0.CO;2-S

[B5] Young A de Oliveira Salles P Lim S Cohen C Petros J Marshall F Neish A Amin M Beta defensin-1, parvalbumin, and vimentin: a panel of diagnostic immunohistochemical markers for renal tumors derived from gene expression profiling studies using cDNA microarrays Am J Surg Pathol 2003 27 199 205 10.1097/00000478-200302000-00008 12548166

[B6] Donald C Sun C Lim S Macoska J Cohen C Amin M Young A Ganz T Marshall F Petros J Cancer-specific loss of beta-defensin 1 in renal and prostatic carcinomas Lab Invest 2003 83 501 505 12695553 10.1097/01.lab.0000063929.61760.f6

[B7] Eisenhauer PB Lehrer RI Mouse neutrophils lack defensins Infect Immun 1992 60 3446 3447 1639513 10.1128/iai.60.8.3446-3447.1992 PMC257335

[B8] Ouellette AJ Greco RM James M Frederick D Naftilan J Fallon JT Developmental regulation of cryptdin, a corticostatin/defensin precursor mRNA in mouse small intestinal crypt epithelium J Cell Biol 1989 108 1687 1695 10.1083/jcb.108.5.1687 2715173 PMC2115551

[B9] Ouellette AJ Lualdi JC A novel mouse gene family coding for cationic, cysteine-rich peptides. Regulation in small intestine and cells of myeloid origin [published erratum appears in J Biol Chem 1994 Jul 15;269(28):18702] J Biol Chem 1990 265 9831 9837 2351676

[B10] Ouellette AJ Lauldi JC A novel gene family coding for cationic, cysteine-rich peptides. Regulation in mouse small intestine and cells of myeloid origin J Biol Chem 1994 269 18702 8034619

[B11] Ouellette AJ Pravtcheva D Ruddle FH James M Localization of the cryptdin locus on mouse chromosome 8 Genomics 1989 5 233 239 10.1016/0888-7543(89)90051-7 2571573

[B12] Ouellette AJ Pravtcheva D Ruddle F James M Erratum Genomics 1992 12 626 10.1016/0888-7543(92)90462-2 2571573

[B13] Lin MY Munshi IA Ouellette AJ The defensin-related murine CRS1C gene: Expression in paneth cells and linkage to Defcr, the cryptdin locus Genomics 1992 14 363 368 10.1016/S0888-7543(05)80227-7 1427853

[B14] Huttner KM Selsted ME Ouellette AJ Structure and Diversity of the Murine Cryptdin Gene Family Genomics 1994 19 448 453 10.1006/geno.1994.1093 8188287

[B15] Huttner KM Selsted ME Ouellette AJ Erratum - Structure and diversity of the murine cryptdin gene family Genomics 1995 25 762 10.1016/0888-7543(95)80029-L 8188287

[B16] Ouellette AJ Miller SI Henschen AH Selsted ME Purification and primary structure of murine cryptdin-1, a Paneth cell defensin FEBS Letters 1992 304 146 148 10.1016/0014-5793(92)80606-H 1618314

[B17] Selsted ME Miller SI Henschen AH Ouellette AJ Enteric defensins: antibiotic peptide components of intestinal host defense J Cell Biol 1992 118 929 936 10.1083/jcb.118.4.929 1500431 PMC2289569

[B18] Ganz T Defensins: antimicrobial peptides of innate immunity Nat Rev Immunol 2003 3 710 720 10.1038/nri1180 12949495

[B19] Lehrer RI Ganz T Defensins of vertebrate animals Current Opinion in Immunology 2002 14 96 102 10.1016/S0952-7915(01)00303-X 11790538

[B20] Ouellette AJ Defensin-mediated innate immunity in the small intestine Best Pract Res Clin Gastroenterol 2004 18 405 419 10.1016/j.bpg.2003.10.010 15123078

[B21] Nguyen TX Cole AM Lehrer RI Evolution of primate [theta]-defensins: a serpentine path to a sweet tooth Peptides 2003 24 1647 1654 10.1016/j.peptides.2003.07.023 15019196

[B22] Zou J Mercier C Koussounadis A Secombes C Discovery of multiple beta-defensin like homologues in teleost fish Molecular Immunology 2007 44 638 647 10.1016/j.molimm.2006.01.012 16540171

[B23] Patil A Hughes AL Zhang G Rapid evolution and diversification of mammalian {alpha}-defensins as revealed by comparative analysis of rodent and primate genes Physiol Genomics 2004 20 1 11 10.1152/physiolgenomics.00150.2004 15494476

[B24] Semple CA Gautier P Taylor K Dorin JR The changing of the guard: Molecular diversity and rapid evolution of β-defensins Molecular Diversity 2006 10 575 584 10.1007/s11030-006-9031-7 16969721

[B25] Radhakrishnan Y Fares MA French FS Hall SH Comparative genomic analysis of a mammalian {beta}-defensin gene cluster Physiol Genomics 2007 30 213 222 10.1152/physiolgenomics.00263.2006 17456736

[B26] Lynn DJ Lloyd AT Fares MA O'Farrelly C Evidence of Positively Selected Sites in Mammalian {alpha}-Defensins Mol Biol Evol 2004 21 819 827 10.1093/molbev/msh084 14963090

[B27] Hall SH Yenugu S Radhakrishnan Y Avellar MCW Petrusz P French FS Characterization and functions of beta defensins in the epididymis Asian J Androl 2007 9 453 462 10.1111/j.1745-7262.2007.00298.x 17589782

[B28] Patil AA Cai Y Sang Y Blecha F Zhang G Cross-species analysis of the mammalian {beta}-defensin gene family: presence of syntenic gene clusters and preferential expression in the male reproductive tract Physiol Genomics 2005 23 5 17 10.1152/physiolgenomics.00104.2005 16033865

[B29] Church DM Goodstadt L Hillier LW Zody MC Goldstein S She X Bult CJ Agarwala R Cherry JL DiCuccio M Hlavina W Kapustin Y Meric P Maglott D Birtle Z Marques AC Graves T Zhou S Teague B Potamousis K Churas C Place M Herschleb J Runnheim R Forrest D Amos-Landgraf J Schwartz DC Cheng Z Lindblad-Toh K Eichler EE Ponting CP The Mouse Genome Sequencing C Lineage-Specific Biology Revealed by a Finished Genome Assembly of the Mouse PLoS Biol 2009 7 e1000112 10.1371/journal.pbio.1000112 19468303 PMC2680341

[B30] Huttner KM Ouellette AJ A Family of Defensin-like Genes Codes for Diverse Cysteine-Rich Peptides in Mouse Paneth Cells Genomics 1994 24 99 109 10.1006/geno.1994.1586 7896294

[B31] Hornef M Putsep K Karlsson J Refai E Andersson M Increased diversity of intestinal antimicrobial peptides by covalent dimer formation Nat Immunol 2004 5 836 843 10.1038/ni1094 15235601

[B32] Jalkanen J Huhtaniemi I Poutanen M Discovery and characterization of new epididymis-specific beta-defensins in mice Biochim Biophys Acta 2005 1730 22 30 10.1016/j.bbaexp.2005.05.010 16023745

[B33] Tsutsumi-Ishii Y Hasebe T Nagaoka I Role of CCAAT/Enhancer-Binding Protein Site in Transcription of Human Neutrophil Peptide-1 and -3 Defensin Genes J Immunol 2000 164 3264 3273 10706719 10.4049/jimmunol.164.6.3264

[B34] Landry CR Lemos B Rifkin SA Dickinson WJ Hartl DL Genetic Properties Influencing the Evolvability of Gene Expression Science 2007 317 118 121 10.1126/science.1140247 17525304

[B35] Zheng D Frankish A Baertsch R Kapranov P Reymond A Choo SW Lu Y Denoeud F Antonarakis SE Snyder M Ruan Y Wei C-L Gingeras TR Guigo R Harrow J Gerstein MB Pseudogenes in the ENCODE regions: Consensus annotation, analysis of transcription, and evolution Genome Res 2007 17 839 851 10.1101/gr.5586307 17568002 PMC1891343

[B36] Piehler A Hellum M Wenzel J Kaminski E Haug K Kierulf P Kaminski W The human ABC transporter pseudogene family: Evidence for transcription and gene-pseudogene interference BMC Genomics 2008 9 165 10.1186/1471-2164-9-165 18405356 PMC2329642

[B37] Blake JA Bult CJ Eppig JT Kadin JA Richardson JE the Mouse Genome Database G The Mouse Genome Database genotypes::phenotypes Nucleic Acids Res 2008 37 D712 719 10.1093/nar/gkn886 18981050 PMC2686566

[B38] Jing W Hunter HN Tanabe H Ouellette AJ Vogel HJ Solution Structure of Cryptdin-4, a Mouse Paneth Cell α-Defensin Biochemistry 2004 43 15759 15766 10.1021/bi048645p 15595831

[B39] Shirafuji Y Tanabe H Satchell DP Henschen-Edman A Wilson CL Ouellette AJ Structural determinants of procryptdin recognition and cleavage by matrix metalloproteinase-7 Journal of Biological Chemistry 2002 278 7910 7919 10.1074/jbc.M210600200 12482850

[B40] UCSC Genome Browser http://genome.ucsc.edu/cgi-bin/hgGateway

[B41] Mouse Chromosome 8 Linkage Map http://www.informatics.jax.org/searches/linkmap_form.shtml

[B42] Lynn DJ Higgs R Lloyd AT O'Farrelly C Herve-Grepinet V Nys Y Brinkman FSL Yu P-L Soulier A Kaiser P Zhang G Lehrer RI Avian beta-defensin nomenclature: A community proposed update Immunology Letters 2007 110 86 89 10.1016/j.imlet.2007.03.007 17467809

[B43] Schutte BC Mitros JP Bartlett JA Walters JD Jia HP Welsh MJ Casavant TL McCray PB Erratum - Discovery of five conserved β-defensin gene clusters using a computational search strategy PNAS 2002 99 14611 10.1073/pnas.042692699 PMC122330 11854508

[B44] Lynn DJ Bradley DG Discovery of alpha-defensins in basal mammals Dev Comp Immunol 2007 31 963 967 10.1016/j.dci.2007.01.007 17367857

[B45] Genome Reference Consortium http://www.ncbi.nlm.nih.gov/projects/genome/assembly/grc/index.shtml

[B46] Hollox E Armour J Barber J Extensive normal copy number variation of a beta-defensin antimicrobial-gene cluster Am J Hum Genet 2003 73 591 600 10.1086/378157 12916016 PMC1180683

[B47] Bakar SA Hollox EJ Armour JAL Allelic recombination between distinct genomic locations generates copy number diversity in human β-defensins PNAS 2009 106 853 858 10.1073/pnas.0809073106 19131514 PMC2630076

[B48] Linzmeier RM Ganz T Human defensin gene copy number polymorphisms: Comprehensive analysis of independent variation in [alpha]- and [beta]-defensin regions at 8p22-p23 Genomics 2005 86 423 430 10.1016/j.ygeno.2005.06.003 16039093

[B49] Potter SC Clarke L Curwen V Keenan S Mongin E Searle SMJ Stabenau A Storey R Clamp M The Ensembl Analysis Pipeline Genome Res 2004 14 934 941 10.1101/gr.1859804 15123589 PMC479123

[B50] Benson G Tandem repeats finder: a program to analyze DNA sequences Nucleic Acids Res 1999 27 573 580 10.1093/nar/27.2.573 9862982 PMC148217

[B51] Mott R EST_GENOME: a program to align spliced DNA sequences to unspliced genomic DNA Comput Appl Biosci 1997 13 477 478 9283765 10.1093/bioinformatics/13.4.477

[B52] Birney E Clamp M Durbin R GeneWise and Genomewise Genome Res 2004 14 988 995 10.1101/gr.1865504 15123596 PMC479130

[B53] Burge C Karlin S Prediction of complete gene structures in human genomic DNA Journal of Molecular Biology 1997 268 78 94 10.1006/jmbi.1997.0951 9149143

[B54] Lowe T Eddy S tRNAscan-SE: a program for improved detection of transfer RNA genes in genomic sequence Nucleic Acids Res 1997 25 955 964 10.1093/nar/25.5.955 9023104 PMC146525

[B55] Down TA Hubbard TJP Computational Detection and Location of Transcription Start Sites in Mammalian Genomic DNA Genome Res 2002 12 458 461 10.1101/gr.216102 11875034 PMC155284

[B56] Searle SMJ Gilbert J Iyer V Clamp M The Otter Annotation System Genome Res 2004 14 963 970 10.1101/gr.1864804 15123593 PMC479127

[B57] Sonnhammer E Wootton J Integrated graphical analysis of protein sequence features predicted from sequence composition Proteins 2001 45 262 273 10.1002/prot.1146 11599029

