# Supplementary material for: Manual annotation and analysis of the defensin gene cluster in the C57BL/6J mouse reference genome
Source: BMC Genomics. 2009 Dec 15;10:606. doi: 10.1186/1471-2164-10-606 (PMC2807441; doi:10.1186/1471-2164-10-606)
Supplement: Additional file 1 — Supplemental Tables [file 1471-2164-10-606-S1.DOC]

**Supplemental Table S1:**

| **Organism /**  **species** | **Gene Symbol** | **Gene name** | **Proposed gene symbol** | **Exon count** | **Vega-Gene ID** | **TATA-Box** | **TATA-Box genomic coordinates** | **Polymorphic / CNV / duplication** | **Human orthologue** | **Rat**  **Orthologue (potential)** |
| --- | --- | --- | --- | --- | --- | --- | --- | --- | --- | --- |
| Mouse chr8 C57BL/6J | Defb40 | defensin beta 40 |  | 2 | OTTMUSG00000020772 | - |  |  |  | Defb40 |
| Mouse chr8 C57BL/6J | Defb37 | defensin beta 37 |  | 2 | OTTMUSG00000020771 | - |  |  |  | Defb37 |
| Mouse chr8 C57BL/6J | Defb38 | defensin beta 38 |  | 2 | OTTMUSG00000020768 | - |  |  |  | Defb38 |
| Mouse chr8 C57BL/6J | Defb39 | defensin beta 39 |  | 2 | OTTMUSG00000020770 | - |  |  |  | Defb39 |
| Mouse chr8 C57BL/6J | Defb12 | defensin beta 12 |  | 3 | OTTMUSG00000020595 | TATAAATG | 19114860 - 19114865  (strand -1) |  | DEFB105 (MGI & ref) | Defb12 |
| Mouse chr8 C57BL/6J | Defb34 | defensin beta 34 |  | 2 | OTTMUSG00000020594 | - |  |  |  | n/a |
| Mouse chr8 C57BL/6J | AC116394.3 | sperm-associated antigen 11c/h (Spag11c/h) |  | 2 and 3 | OTTMUSG00000020593 |  |  |  |  | NP_001032941.1  (20 aa shorter – deletion) |
| Mouse chr8 C57BL/6J | Spag11 | sperm associated antigen 11 |  | 2 | OTTMUSG00000020599 | TATAAATG | 19157855 - 19157862 |  | SPAG11B | Spag11 |
| Mouse chr8 C57BL/6J | Defb14 | defensin beta 14 |  | 2 | OTTMUSG00000020600 | - |  |  | DEFB103 (MGI & ref) | Defb14 |
| Mouse chr8 C57BL/6J | Defb4 | defensin beta 4 |  | 2 | OTTMUSG00000020596 | - |  |  | DEFB4 (ref) | Defb4 |
| Mouse chr8 C57BL/6J | Defb6 | defensin beta 6 |  | 2 | OTTMUSG00000020598 |  |  |  |  | n/a |
| Mouse chr8 C57BL/6J | AC163997.1 | beta-defensin 53 (Defb53) | Defb# | 2 | OTTMUSG00000020619 | - |  |  |  | n/a |
| Mouse chr8 C57BL/6J | Defb5 | defensin beta 5 |  | 2 | OTTMUSG00000020669 | - |  |  |  | n/a |
| Mouse chr8 C57BL/6J | Defb3 | defensin beta 3 |  | 2 | OTTMUSG00000020668 | - |  |  |  | Defb3 |
| Mouse chr8 C57BL/6J | Defb8 | defensin beta 8 |  | 2 | OTTMUSG00000020716 | - |  |  |  | n/a |
| Mouse chr8 C57BL/6J | Defb7 | defensin beta 7 |  | 2 | OTTMUSG00000020722 | - |  |  |  | n/a |
| Mouse chr8 C57BL/6J | AC140205.1 | CRS4C-6 (cryptdin-related sequence peptide) | Defa-rs# | 2 | OTTMUSG00000018344 | TATAAATG | 22084456 - 22084463  (strand -1) |  |  | n/a |
| Mouse chr8 C57BL/6J | AC140205.2 | Beta-defensin 51 (Defb51) | Defb# | 3 | OTTMUSG00000018889 | - |  |  |  | Defb51 |
| Mouse chr8  C57BL/6J | AC140205.3 | beta-defensin 52 (Defb52) | Defb# | 4 | OTTMUSG00000018888 | - |  |  |  | Defb52 |
| Mouse chr8 C57BL/6J | AC140205.4 | beta-defensin 33 (Defb33) | Defb# | 3 | OTTMUSG00000018925 | - |  |  |  | Defb33 |
| Mouse chr8 C57BL/6J | Defcr21 | defensin related cryptdin 21 | **Defa21** | 2 | OTTMUSG00000019489 | TATAAATA | 22165193 - 22165200 |  |  | n/a |
| Mouse chr8 C57BL/6J | Defcr23 | defensin related cryptdin 23 | **Defa23** | 2 | OTTMUSG00000019488 | TATAAATG | 22194686 - 22194693 |  |  | n/a |
| Mouse chr8 C57BL/6J | AC129197.1 | novel protein similar to defensin related cryptdin 5 | Defa5[suffix] | 2 | OTTMUSG00000018258 | TATAAATG | 22204671 - 22204678 |  |  | n/a |
| Mouse chr8 C57BL/6J | Defcr25 | defensin related cryptdin 25 | **Defa25** (Defcr2 name should be removed) | 2 | OTTMUSG00000019700 |  |  |  |  | n/a |
| Mouse chr8 C57BL/6J | AC129197.2 | novel defensin related sequence cryptdin peptide CRS1C | Defa-rs2 | 2 | OTTMUSG00000018260 | TATAAATG | 22235796 22235803  (strand -1) |  |  | n/a |
| Mouse chr8 C57BL/6J | AC166039.1 | novel defensin related cryptdin | Defa# | 2 | OTTMUSG00000019742 | TATAAAGG | 22274268 - 22274275 |  |  | n/a |
| Mouse chr8 C57BL/6J | **AC166039.2**  **Defcr22** | novel defensin related cryptdin | **Defa22** | 2 | OTTMUSG00000019763 | TATAAATA | 22301925 - 22301932 |  |  | n/a |
| Mouse chr8 C57BL/6J | AC166039.3 | novel defensin related cryptdin **identical to Defcr23** | Defa23[suffix] | 2 | OTTMUSG00000019762 | TATAAATG | 22331219 - 22331226 | Yes |  | n/a |
| Mouse chr8 C57BL/6J | AC129174.1 | novel defensin related cryptdin | Defa# | 2 | OTTMUSG00000019786 | TATAAATG | 22341209 - 22341216 |  |  | n/a |
| Mouse chr8 C57BL/6J | AC129174.4 | novel defensin related cryptdin | Defa# | 2 | OTTMUSG00000019784 | - |  |  |  | n/a |
| Mouse chr8 C57BL/6J | Defcr3 | defensin related cryptdin 3 | **Defa3** | 2 | OTTMUSG00000019782 | TATAAATG | 22427048 - 22427055 |  |  | n/a |
| Mouse chr8 C57BL/6J | AC129174.7 | novel protein similar to defensin related cryptdin 5 | **Defa5**[suffix] | 2 | OTTMUSG00000019785 | TATAAATG | 22437044 - 22437051 |  |  | n/a |
| Mouse chr8 C57BL/6J | **Defcr-rs1**  (alias  CRS1C-2) | Defensin related sequence cryptdin peptide | **Defa-rs1** | 2 | OTTMUSG00000019792 | TATAAATG | 22466721 - 22466728  (strand -1) |  |  | n/a |
| Mouse chr8 C57BL/6J | AC133094.3 | novel defensin related cryptdin | Defa# | 2 | OTTMUSG00000019857 | - |  |  |  | n/a |
| Mouse chr8 C57BL/6J | AC133094.5 | novel defensin related sequence cryptdin peptide CRS1C | Defa-rs4 | 2 | OTTMUSG00000019859 | TATAAATG | 22567056 - 22567063  (strand -1) | Yes, identical to AC133094.13 |  | n/a |
| Mouse chr8 C57BL/6J | Defcr20 | defensin related cryptdin 20 | **Defa20** | 2 | OTTMUSG00000019856 | TATAAATG | 22619699 - 22619706 |  |  | n/a |
| Mouse chr8 C57BL/6J | AC133094.9 | novel defensin related cryptdin **identical to Defcr20** | Defa20[suffix] | 2 | OTTMUSG00000019860 | TATAAATG | 22639473 - 22639480 | Yes |  | n/a |
| Mouse chr8 C57BL/6J | AC133094.1 | novel protein similar to defensin related cryptdin 5 |  | 2 | OTTMUSG00000018259 | TATAAATG | 22675812 - 22675819 | Yes |  | n/a |
| Mouse chr8 C57BL/6J | AC133094.11 | novel defensin related cryptdin |  | 2 | OTTMUSG00000019896 | - |  |  |  | n/a |
| Mouse chr8 C57BL/6J | AC133094.13 | novel defensin related sequence cryptdin peptide CRS1C | Defcr-rs5[suffix] | 2 | OTTMUSG00000019893 | TATAAATG | 22705004 - 22705011  (strand -1) | Yes, identical to AC133094.5 |  | n/a |
| Mouse chr8 C57BL/6J | Defcr26 | defensin related cryptdin 26 | **Defa26** | 2 | OTTMUSG00000019889 | - |  |  |  | n/a |
| Mouse chr8 C57BL/6J | AC134533.2 | novel defensin related cryptdin **identical to Defcr3** | Defa3[suffix] | 2 | OTTMUSG00000019892 | TATAAATG | 22766199 - 22766206 | Yes |  | n/a |
| Mouse chr8 C57BL/6J | AC134533.3 | novel protein similar to defensin related cryptdin 5 | Defa5[suffix] | 2 | OTTMUSG00000019924 | TATAAATG | 22776195 - 22776202 | Yes |  | n/a |
| Mouse chr8 C57BL/6J | AC134533.6 | novel defensin related cryptdin (CRS1C-3) | Defa-rs3 | 2 | OTTMUSG00000019927 | TATAAATG | 22814141 - 22814148  (strand -1) |  |  | n/a |
| Mouse chr8 C57BL/6J | Defcr24 | defensin related cryptdin 24 | **Defa24** | 2 | OTTMUSG00000019980 | TATAAATG | 22844935 - 22844942 |  |  | Defcr24 |
| Mouse chr8 C57BL/6J | Defb1 | defensin beta 1 |  | 2 | OTTMUSG00000019983 | TATAAAAA | 22887029 - 22887036 |  | DEFB1 (MGI & ref) | Defb1 |
| Mouse chr8 C57BL/6J | Defb50 | defensin beta 50 |  | 2 | OTTMUSG00000019981 | TATAAATC | 22933984 - 22933991 |  |  | Defb50 |
| Mouse chr8 C57BL/6J | Defb2 | defensin beta 2 |  | 2 | OTTMUSG00000020785 | - |  |  |  | Defb2 |
| Mouse chr8 C57BL/6J | Defb10 | defensin beta 10 |  | 2 | OTTMUSG00000020783 | - |  |  |  | Defb9  53%  Defb10  53% |
| Mouse chr8 C57BL/6J | Defb9 | defensin beta 9 |  | 2 | OTTMUSG00000020782 | - |  |  |  | Defb11  53%  questionable |
| Mouse chr8 C57BL/6J | Defb11 | defensin beta 11 |  | 2 | OTTMUSG00000020784 | - |  |  |  | Defb9  50%  Defb10  50%  Both questionable |
| Mouse chr8 C57BL/6J | Defb15 | defensin beta 15 |  | 2 | OTTMUSG00000020830 | TATAAAGG | 23057269 -  23057262  (strand -1) |  | DEFB106 (MGI & ref) | Defb15 (syntenic)  NP_001032609.1 (12 aa longer; not syntenic)  Both questionable |
| Mouse chr8 C57BL/6J | Defb35 | defensin beta 35 |  | 2 | OTTMUSG00000020829 | - |  |  |  | n/a |
| Mouse chr8 C57BL/6J | Defb13 | defensin beta 13 |  | 2 | OTTMUSG00000020827 |  |  |  | DEFB107 (ref) | Defb13  75%  questionable |

Approved gene symbols are shown in bold.

“AC” gene symbols are provisional and based on their position on the clone.

9 alpha-defensins in rat without mouse orthologs.

n/a = corresponding rat gene not found

**Supplemental Table S2:**

| **Clone** | **OTTMUSG** | **MGI name** | **Pseudogene name** | **Pseudogene Type** | **Organism** |
| --- | --- | --- | --- | --- | --- |
| AC140205.9 | AC140205.9 |  | defensin pseudogene similar to Defa7 | Unprocessed | Mouse |
| AC129197 | OTTMUSG00000019818 | Defa-ps3 | defensin related cryptdin pseudogene | Unprocessed | Mouse |
| AC166039 | OTTMUSG00000019815 | Defa-ps4 | pseudogene similar to part of defensin related cryptdin | - | Mouse |
| AC129174 | OTTMUSG00000019783 | Defa-ps5 | novel defensin related cryptdin pseudogene | Unprocessed | Mouse |
|  | OTTMUSG00000019780 | Defa-ps6 | defensin, alpha, pseudogene 1 | Transcribed_unprocessed | Mouse |
|  | OTTMUSG00000019817 | Defa-ps7 | pseudogene similar to part of defensin related cryptdin | - | Mouse |
|  | OTTMUSG00000019781 |  | novel defensin related cryptdin pseudogene | Unprocessed | Mouse |
|  | OTTMUSG00000019794 |  | novel defensin related cryptdin pseudogene | Unprocessed | Mouse |
|  | OTTMUSG00000019793 |  | novel defensin related cryptdin pseudogene | Unprocessed | Mouse |
|  | OTTMUSG00000019795 |  | pseudogene similar to part of defensin related cryptdin | Pseudogene | Mouse |
| AC133094 | OTTMUSG00000019855 |  | defensin related cryptdin pseudogene | Unprocessed | Mouse |
|  | OTTMUSG00000019858 |  | pseudogene similar to part of defensin related cryptdin | Pseudogene | Mouse |
|  | OTTMUSG00000019890 |  | defensin alpha pseudogene | Unprocessed | Mouse |
| AC134533 | OTTMUSG00000019894 |  | defensin alpha pseudogene | Unprocessed | Mouse |
|  | OTTMUSG00000019925 | Defa-ps9 | defensin alpha pseudogene | Unprocessed | Mouse |
|  | OTTMUSG00000019923 | Defa-ps1 | defensin alpha pseudogene **identical to Defa-ps1** | Transcribed_unprocessed | Mouse |
|  | OTTMUSG00000019929 | Defa-ps10 | pseudogene similar to part of defensin related cryptdin | Pseudogene | Mouse |
|  | OTTMUSG00000019982 | Defa-ps11 | defensin alpha pseudogene | Unprocessed | Mouse |
| AC116394 | OTTMUSG00000020597 | Defa-ps12 | defensin beta 46 pseudogene | Unprocessed | Mouse |
| AC113099 | OTTMUSG00000020671 | Defa-ps13 | defensin beta 54 pseudogene (Defb54-ps) | Unprocessed | Mouse |
|  | OTTMUSG00000020719 | Defa-ps14 | novel defensin alpha pseudogene | Unprocessed | Mouse |
| AC121131 | OTTMUSG00000020718 | Defa-ps15 | novel defensin pseudogene | Unprocessed | Mouse |

**Supplemental Table S3:**

**Supplemental Table S4: Summary of Best Non-mouse Hits for CRS BLAST**

**Supplemental Table S5: Genome Browser Comparison of mouse alpha-defensin genes**

Vega-annotated mouse alpha-defensin genes were used to query Ensembl (v.50), MGI (v.4.11) and NCBI (Build 37.1) databases. Both Vega gene symbols and Otter IDs were used because, depending on the database and gene, different results were sometimes returned with either search. As well, not all databases recognize the Vega accession number if a gene name hasn’t been assigned. A gene linked to Vega indicates that there is a reference to the Vega Gene/ID as being the same gene (i.e. mapped to the same position on the chromosome), but has been given a database-specific name. If the database had referenced another database to obtain the Otter ID but there is no acknowledgement to the mapping in Vega, the gene was not considered linked and put into the Additional Gene column. Genes that were identified in the searches but were different to the Vega gene were also put into the Additional Gene column. NCBI was searched with Otter IDs and gene name because some Vega accession numbers return BAC clones in the results. NCBI does not cross-reference Vega, rather they obtain their information from MGI, so none of the genes are directly linked to Vega; genes linked to the Vega-linked Ensembl genes are therefore listed.

**Supplemental Table S6: Defensin genes currently missing from the mouse reference genome**

**Supplemental Table S7: Defensin nomenclature schemes**
