# Supplementary material for: Manual annotation and analysis of the defensin gene cluster in the C57BL/6J mouse reference genome
Source: BMC Genomics. 2009 Dec 15;10:606. doi: 10.1186/1471-2164-10-606 (PMC2807441; doi:10.1186/1471-2164-10-606)
Supplement: Additional file 2 — Supplemental Information [file 1471-2164-10-606-S2.DOC]

**Supplemental Information**

***S1. CRS Family – Unique to Mouse?***

As we have annotated three novel CRS genes and the expression of this family of defensin-like peptides has only been reported in mice, it was of interest to determine whether other species, rat in particular, have homologous peptides. Literature and databases were searched for annotated or experimental evidence of genes and peptides to identify novel rat peptide sequences homologous to that of the CRS family. Referring to the literature, the CRS family cluster separately from other mammalian alpha-defensins and it is argued that the mouse CRS family and rat alpha-defensins have evolved separately with a common gene ancestor prior to speciation [1].

Pfam 22.0 was queried using the batch search option with default parameters. For all of the CRS peptides, the only significant hit (based on e-value) is the defensin_propep domain, which is general and not species specific. Individual searches returned specific defensins but none align at the C-terminal end of the CRS peptides. Extensive BLAST searches using both genomic and peptide CRS sequences suggest rat alpha-defensins as potential homologues. However results of all BLAST searches, including those against unannotated databases, show significant hits to the 5’ and N-terminal region of the genomic and peptide sequences, respectively, but poor matches to 3’ and C-terminal regions. Results can be viewed in Additional file 1: Supplemental Tables S3&S4 and Additional file 3: Supplemental Figure S1. An additional protein alignment of mouse CRS and rat alpha-defensin peptides (Additional file 3: Supplemental Figure S2) confirms this lack of homology. At this time it appears the CRS family is unique to mouse.

***S2. Conserved synteny in gaps proximal to defensins***

Interspecies comparison is important not only from a biological perspective, but also to ascertain whether the human and/or rat genome assemblies can be used to facilitate closing of the estimated 2 Mb gap of mouse Chromosome 8, adjacent to the two alpha-defensin loci (Additional file 3: Supplemental Figure S5; 8: 18,908,000 – 23,400,000 bp). A recent study has compared the mouse NCBI Build 36 to an optical map [2]; their results show that this gap is probably smaller than the estimated 2 Mb, however its size still could not be assessed precisely.

Ensembl v.49 was used to display regions of conserved synteny between the mouse, human and rat alpha-defensin regions. The conserved syntenic region of the human genome is Chromosome 8p23.1. Additional file 3: Supplemental Figure S6 shows the arrangement of the human defensin genes within this region (8: 6,600,000 – 8,100,000 bp), which includes one gap approximately 100 kb in size. This region is also flanked at the 5’ end by *XKR5*, the homologue of the mouse *Xkr5*. However, whereas in the mouse *Ccdc70*, *Atp7b* and *Alg11* are found 3' to the defensin gene cluster on Chromosome 8, *CCDC70* does not flank the 3’ end in human, but rather is located on human Chromosome 13q14.3 where the following two telomeric gene homologues *ATP7B* and *ALG11* have also been mapped. There are no defensin genes in this region of human Chromosome 13, which indicates that the breakpoint occurred telomeric to the defensin cluster and a survey of this region shows a complete assembly without any gaps.

The quality of the human genome assembly near the defensin regions appears to be better than that of mouse. This may be due to a larger number of defensin genes within the mouse genome as compared to the human genome, as well as the high similarity between the mouse alpha-defensin genes in particular. To investigate this further we used Ensembl to analyze the conserved syntenic regions of the rat genome compared to the mouse. Additional file 3: Supplemental Figure S7 shows arrangement of alpha-defensins, as well as some beta-defensins and other genes within that conserved syntenic locus (16: 73,700,000 – 75,800,000 bp). Nine rat alpha-defensin genes and one defensin-related gene are located on Chromosome 16q12.5. There are a few gaps in the region near to the defensin genes, one in particular of about 120 kb in the middle of the alpha- and beta-defensin cluster, and another 5’ to the defensin region about 200 kb in size. The assembly of the rat reference sequence appears to be more similar to that of the human sequence compared to the mouse sequence with respect to the level of completion of defensin-rich regions. However it is important to note that the rat genome sequence is a draft sequence which differs with respect to the finished sequence of both human and mouse genomes [3]. The approach for the sequencing of the rat genome was a combination of whole genome shotgun (WGS) and bacterial artificial chromosome (BAC) sequencing, and the authors argue that this approach has generated sequence with quality near to that of finished sequence [3]. Another caveat is that the human and mouse genomes aided the assembly of the rat genome in difficult regions [3], therefore any errors in either the human and mouse assemblies within defensin regions could translate into errors in the rat genome. A coordinated effort has been undertaken for the generation of a new rat genome build and the anticipated release is imminent [4]. Re-examination of the defensin clusters will determine whether our analyses and observations hold true for these regions, in particular.

Similar to the annotation of mouse alpha-defensins only on Chromosome 8, human and rat alpha-defensins have only been identified on Chromosome 8 and 16, respectively. This is in contrast to the presence of human, rat and mouse beta-defensins on multiple chromosomes (human – Chromosomes 6p21, 8p23.1, 20q11.1 and 20p13, rat – 3q41, 9q13, 15p12 and 16q12.5 and mouse – 1A4, 2H1, 8A3 and 14D1). The assembly of human Chromosomes 6p21 and 20p13 are complete with no gaps, but there is a gap 5’ to the beta-defensin cluster on 20q11.1; however this gap is also near to the centromere, which was not targeted by the genome projects due to the difficulty in sequencing highly repetitive alpha-satellite DNA in heterchromatic regions [5, 6]. Additional rat beta-defensins are located on Chromosomes 3q41, 9q13, 15p12 and 16q12.5; these regions appear complete, with the exception of 3q41, which has an 11kb gap within the beta-defensin cluster and 16q12.5, which contains both alpha- and beta-defensins genes, as previously described.

The assemblies of the mouse, rat and human genomes are more complete near regions of beta-defensins compared to alpha-defensins since beta-defensins are not as genetically similar as alpha-defensins. Beta-defensins have had more time for movement associated with chromosomal rearrangements and multiple duplication events as compared to alpha-defensins; however mouse alpha-defensins have undergone a rapid expansion that has not occurred to the same extent in human and rat. Rapidly changing regions are interesting in evolutionary terms but are difficult to assemble into finished sequence [3], and additional defensin genes may be present within gaps in the assembly. These factors reinforce the biological importance and need for further characterization.

***S3. Copy number polymorphisms and expression level variation detected in human and mouse for the defensin genes***

Several analyses have shown that large-scale copy number polymorphisms are a major source of genetic variation [7-10]. One of these polymorphisms involves the human beta-defensin cluster on 8p23.1. Whereas carriers of a euchromatic variant that is cytogenetically visible have nine to twelve copies of the region [11, 12] most people have two to seven copies [13]. Correlation of beta-defensin copy number with expression levels suggests that variable expression levels could cause different predisposition and susceptibility to infectious diseases. A recent genetic mapping approach confirms two distinct beta-defensin CNV loci, approximately 5Mb apart on human Chromosome band 8p23.1 [14]. The authors state that this contradicts the current genome assembly. As a follow up we analyzed the region surrounding the genomic coordinates indicated in the aforementioned study and found five known beta-defensin genes [15] and one beta-defensin pseudogene (two copies of *Defb130*, *Defb134*, *Defb136* and *Defb137*, data not shown). The relationship between this cluster and the duplicated CNV region is unclear. In a study analyzing the expression levels of human alpha-defensins DEFA1 and DEFA3 a clear correlation could be shown between the relative proportions of DEFA1:DEFA3 mRNA and the corresponding gene numbers [16]. However, combined levels of DEFA1 and DEFA3 were not correlated with gene copy number, indicating influence of trans-acting factors on different expression of both genes [16]. While *DEFA1* copy numbers have been reported to vary between four and 11 in a sample of 111 individuals, the *DEFA3* allele has been absent in 10% of them [16]. In a different study where the absence of *DEFA3* in 697 samples from four different human populations has been tested, the proportion of individuals lacking *DEFA3* is variable from 10% to 37% [17], suggesting differences in innate immune functions between populations. It has been speculated that populations with distinct ecological histories carry different defensin gene copy numbers derived from the selective pressure presented on their historical geographic regions [18]. However a direct correlation between the copy number variation in the defensin region and geographic origin has not been established.

**Supplemental Information References**

1. Patil A, Hughes AL, Zhang G: **Rapid evolution and diversification of mammalian {alpha}-defensins as revealed by comparative analysis of rodent and primate genes**. *Physiol Genomics* 2004, **20**:1-11.

2. Church DM, Goodstadt L, Hillier LW, Zody MC, Goldstein S, She X, Bult CJ, Agarwala R, Cherry JL, DiCuccio M, Hlavina W, Kapustin Y, Meric P, Maglott D, Birtle Z, Marques AC, Graves T, Zhou S, Teague B, Potamousis K, Churas C, Place M, Herschleb J, Runnheim R, Forrest D, Amos-Landgraf J, Schwartz DC, Cheng Z, Lindblad-Toh K, Eichler EE, Ponting CP, The Mouse Genome Sequencing C: **Lineage-specific biology revealed by a finished genome assembly of the mouse**. *PLoS Biol* 2009, **7**:e1000112.

3. Rat Genome Sequencing Project Consortium: **Genome sequence of the brown norway rat yields insights into mammalian evolution**. *Nature* 2004, **428**:493-521.

4. Twigger SN, Pruitt KD, Fernandez-Suarez XM, Karolchik D, Worley KC, Maglott DR, Brown G, Weinstock G, Gibbs RA, Kent J, Birney E, Jacob HJ: **What everybody should know about the rat genome and its online resources**. *Nat Genet* 2008, **40**:523-527.

5. Human Genome Sequencing Consortium International: **Finishing the euchromatic sequence of the human genome**. *Nature* 2004, **431**:931-945.

6. Rudd MK, Willard HF: **Analysis of the centromeric regions of the human genome assembly**. *Trends in Genetics* 2004, **20**:529-533.

7. Freeman JL, Perry GH, Feuk L, Redon R, McCarroll SA, Altshuler DM, Aburatani H, Jones KW, Tyler-Smith C, Hurles ME, Carter NP, Scherer SW, Lee C: **Copy number variation: New insights in genome diversity**. *Genome Res* 2006, **16**:949-961.

8. Sebat J, Lakshmi B, Troge J, Alexander J, Young J, Lundin P, Maner S, Massa H, Walker M, Chi M, Navin N, Lucito R, Healy J, Hicks J, Ye K, Reiner A, Gilliam TC, Trask B, Patterson N, Zetterberg A, Wigler M: **Large-scale copy number polymorphism in the human genome**. *Science* 2004, **305**:525-528.

9. Iafrate A, Feuk L, Rivera M, Listewnik M, Donahoe P, Qi Y, Scherer S, Lee C: **Detection of large-scale variation in the human genome.** *Nat Genet* 2004, **36**:949-951.

10. Fredman D, White S, Potter S, Eichler E, Den Dunnen J, Brookes A: **Complex snp-related sequence variation in segmental genome duplications.** *Nat Genet* 2004, **36**:861-866.

11. Barber J, Joyce C, Collinson M, Nicholson J, Willatt L, Dyson H, Bateman M, Green A, Yates J, Dennis N: **Duplication of 8p23.1: A cytogenetic anomaly with no established clinical significance.** *J Med Genet* 1998, **35**:491-496.

12. O'Malley D, Storto P: **Confirmation of the chromosome 8p23.1 euchromatic duplication as a variant with no clinical manifestations.** *Prenat Diagn* 1999, **19**:183-184.

13. Hollox E, Armour J, Barber J: **Extensive normal copy number variation of a beta-defensin antimicrobial-gene cluster.** *Am J Hum Genet* 2003, **72**:591-600.

14. Bakar SA, Hollox EJ, Armour JAL: **Allelic recombination between distinct genomic locations generates copy number diversity in human β-defensins**. *PNAS* 2009, **106**:853-858.

15. Patil AA, Cai Y, Sang Y, Blecha F, Zhang G: **Cross-species analysis of the mammalian {beta}-defensin gene family: Presence of syntenic gene clusters and preferential expression in the male reproductive tract**. *Physiol Genomics* 2005, **23**:5-17.

16. Aldred PMR, Hollox EJ, Armour JAL: **Copy number polymorphism and expression level variation of the human {alpha}-defensin genes defa1 and defa3**. *Hum Mol Genet* 2005, **14**:2045-2052.

17. Ballana E, Gonzalez J, Bosch N, Estivill X: **Inter-population variability of defa3 gene absence: Correlation with haplotype structure and population variability.** *BMC Genomics* 2007, **8**:1-10.

18. Linzmeier RM, Ganz T: **Human defensin gene copy number polymorphisms: Comprehensive analysis of independent variation in [alpha]- and [beta]-defensin regions at 8p22-p23**. *Genomics* 2005, **86**:423-430.
