# Supplementary material for: Manual annotation and analysis of the defensin gene cluster in the C57BL/6J mouse reference genome
Source: BMC Genomics. 2009 Dec 15;10:606. doi: 10.1186/1471-2164-10-606 (PMC2807441; doi:10.1186/1471-2164-10-606)
Supplement: Additional file 3 — Supplemental Figures [file 1471-2164-10-606-S3.DOC]

**Supplemental Figure S1:** **Alignment of Best Non-mouse Hits for CRS Peptide Sequence BLAST**

1. OTTMUSG00000018260

>UNIPROT:[Q4JEI5_RAT](http://srs.ebi.ac.uk/srsbin/cgi-bin/wgetz?-e+%5Buniprot-id:(Q4JEI5_RAT)%5D|%5Buniprot-acc:(Q4JEI5_RAT)%5D+-noSession) [Q4JEI5](http://www.ebi.ac.uk/interpro/ISpy?ac=Q4JEI5) SubName: Full=Defensin alpha 9 (Length = 103)

Query: 1 MKTXXXXXXXXXXXFQVQADPIQNXXXXXXXXXXXXXXXXAVSVSFGGTEGSALQDVAQR 60

MKT FQ+QADPIQ VSVSF G E SALQ++ +

Sbjct: 1 MKTLVLLSALVLLAFQIQADPIQEAEEETKTEEQPADEDQDVSVSFEGPEPSALQNL-EI 59

Query: 61 RFLWCR 66

R+ W R

Sbjct: 60 RWPWKR 65

Query: 95 CEERQNKTAITTQAPNTQHKGC 116

C +N T+ Q QHK C

Sbjct: 72 CRPYENATSFCAQGLFKQHKFC 93

2. OTTMUSG00000019792

>UNIPROT:[Q4JEI5_RAT](http://srs.ebi.ac.uk/srsbin/cgi-bin/wgetz?-e+%5Buniprot-id:(Q4JEI5_RAT)%5D|%5Buniprot-acc:(Q4JEI5_RAT)%5D+-noSession) [Q4JEI5](http://www.ebi.ac.uk/interpro/ISpy?ac=Q4JEI5) SubName: Full=Defensin alpha 9 (Length = 103)

Query: 1 MKTXXXXXXXXXXXFQVQADPIQNXXXXXXXXXXXXXXXXAVSVSFGGTEGSALQDVAQR 60

MKT FQ+QADPIQ VSVSF G E SALQ++ +

Sbjct: 1 MKTLVLLSALVLLAFQIQADPIQEAEEETKTEEQPADEDQDVSVSFEGPEPSALQNL-EI 59

Query: 61 RFPW 64

R+PW

Sbjct: 60 RWPW 63

Query: 95 CEERQNKTAITTQAPNTQHKGC 116

C +N T+ Q QHK C

Sbjct: 72 CRPYENATSFCAQGLFKQHKFC 93

3. OTTMUSG00000019927

>UNIPROT:[Q4JEI5_RAT](http://srs.ebi.ac.uk/srsbin/cgi-bin/wgetz?-e+%5Buniprot-id:(Q4JEI5_RAT)%5D|%5Buniprot-acc:(Q4JEI5_RAT)%5D+-noSession) [Q4JEI5](http://www.ebi.ac.uk/interpro/ISpy?ac=Q4JEI5) SubName: Full=Defensin alpha 9 (Length = 103)

Query: 1 MKTXXXXXXXXXXXFQVQADPIKNTDXXXXXXXXXXXXDQAVSVSFGGTEGSALQYVAQR 60

MKT FQ+QADPI+ + DQ VSVSF G E SALQ + +

Sbjct: 1 MKTLVLLSALVLLAFQIQADPIQEAEEETKTEEQPADEDQDVSVSFEGPEPSALQNL-EI 59

Query: 61 RFPWCR 66

R+PW R

Sbjct: 60 RWPWKR 65

Query: 95 CKERQNKSAITTQAPNTQHKGC 116

C+ +N ++ Q QHK C

Sbjct: 72 CRPYENATSFCAQGLFKQHKFC 93

4. OTTMUSG00000018344

>UNIPROT:[Q4JEI5_RAT](http://srs.ebi.ac.uk/srsbin/cgi-bin/wgetz?-e+%5Buniprot-id:(Q4JEI5_RAT)%5D|%5Buniprot-acc:(Q4JEI5_RAT)%5D+-noSession) [Q4JEI5](http://www.ebi.ac.uk/interpro/ISpy?ac=Q4JEI5) SubName: Full=Defensin alpha 9 (Length = 103)

Query: 1 MKTXXXXXXXXXXXFYVQADSTQXXXXXXXXXXQPGEEDQGVSVSFEDPERYVLQ 55

MKT F +QAD Q QP +EDQ VSVSFE PE LQ

Sbjct: 1 MKTLVLLSALVLLAFQIQADPIQEAEEETKTEEQPADEDQDVSVSFEGPEPSALQ 55

5. OTTMUSG00000019893

>UNIPROT:[Q4JEI8_RAT](http://srs.ebi.ac.uk/srsbin/cgi-bin/wgetz?-e+%5Buniprot-id:(Q4JEI8_RAT)%5D|%5Buniprot-acc:(Q4JEI8_RAT)%5D+-noSession) [Q4JEI8](http://www.ebi.ac.uk/interpro/ISpy?ac=Q4JEI8) SubName: Full=Defensin alpha 6; (Length = 95)

Query: 1 MKTXXXXXXXXXXXXXXXXDPIQNTDEETKTQEQPGEEDQAVSVSFGGTEGSALQDVAQR 60

MKT DPIQ +EETKT+EQP +EDQ VSVSF G E SALQD

Sbjct: 1 MKTLVLLSALVLVAYQVQADPIQGAEEETKTEEQPSDEDQDVSVSFEGPEASALQDFEIG 60

Query: 61 RFPWCRKCRVCQKC 74

R P R+CR C

Sbjct: 61 R-P-VRRCRCRANC 72

Query: 88 QCPKQPLCKERQNKTAITTQAPNTHHKGC 116

+C + C ++ TA Q P K C

Sbjct: 65 RCRCRANCGPKEYATAFCAQGPFKQFKFC 93

6. OTTMUSG00000019859

>UNIPROT:[Q4JEI8_RAT](http://srs.ebi.ac.uk/srsbin/cgi-bin/wgetz?-e+%5Buniprot-id:(Q4JEI8_RAT)%5D|%5Buniprot-acc:(Q4JEI8_RAT)%5D+-noSession) [Q4JEI8](http://www.ebi.ac.uk/interpro/ISpy?ac=Q4JEI8) SubName: Full=Defensin alpha 6 (Length = 95)

Query: 1 MKTXXXXXXXXXXXXXXXXDPIQNTDEETKTQEQPGEEDQAVSVSFGGTEGSALQDVAQR 60

MKT DPIQ +EETKT+EQP +EDQ VSVSF G E SALQD

Sbjct: 1 MKTLVLLSALVLVAYQVQADPIQGAEEETKTEEQPSDEDQDVSVSFEGPEASALQDFEIG 60

Query: 61 RFPWCRKCRVCQKC 74

R P R+CR C

Sbjct: 61 R-P-VRRCRCRANC 72

Query: 88 QCPKQPLCKERQNKTAITTQAPNTHHKGC 116

+C + C ++ TA Q P K C

Sbjct: 65 RCRCRANCGPKEYATAFCAQGPFKQFKFC 93

**Supplemental** **Figure S2: Alignment of mouse CRS and rat alpha-defensin peptides**


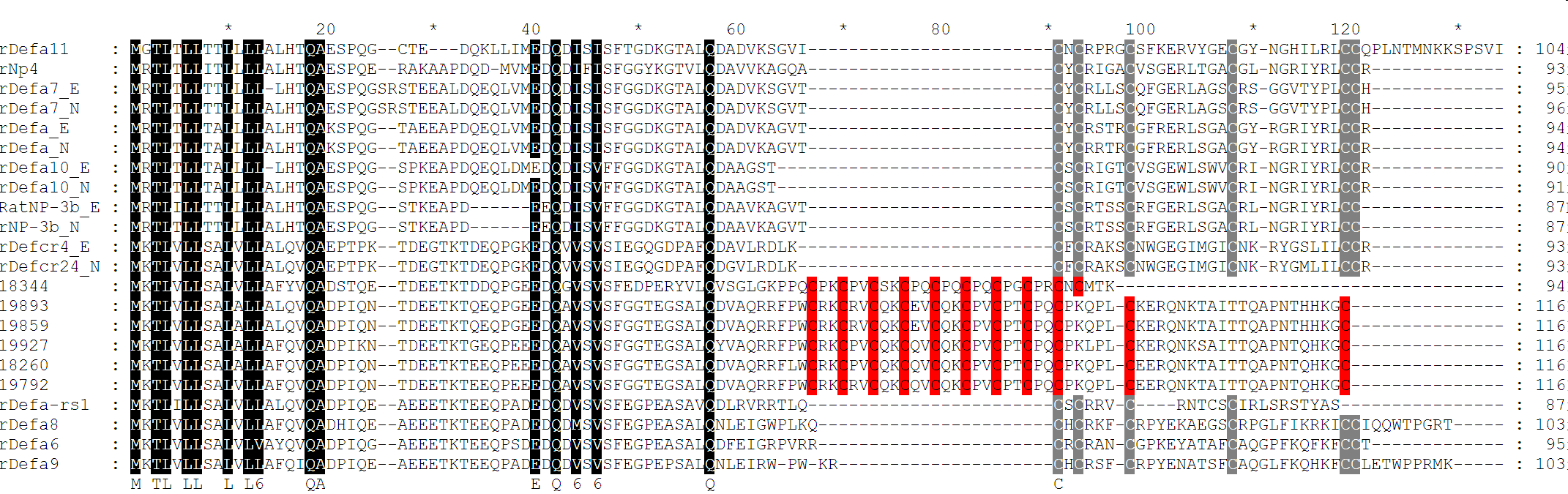
The alignment shows that despite a shared homology in their signal and pro-peptide regions, mouse CRS and rat alpha-defensin peptides are significantly different peptides and none seem to be orthologous even though they most likely share a common ancestor. Mouse sequences are denoted by the last 5 numbers of their Otter ID (e.g. 18344 – OTTMUSG00000018344) and rat sequences are denoted by their gene name preceded by an r (e.g. rDefa11); E and N denote sequences in Ensembl and NCBI, respectively, when the peptides share the same name in both databases but have different peptide sequences. Residues that show 100% conservation across all sequences are highlighted in black. Classical cysteine residues of rat alpha-defensins are highlighted in grey and cysteine residues of the mouse CRS peptides are highlighted in red.

**Supplemental Figure S3: Examples of MGI’s GBrowse for the defensin gene cluster region**


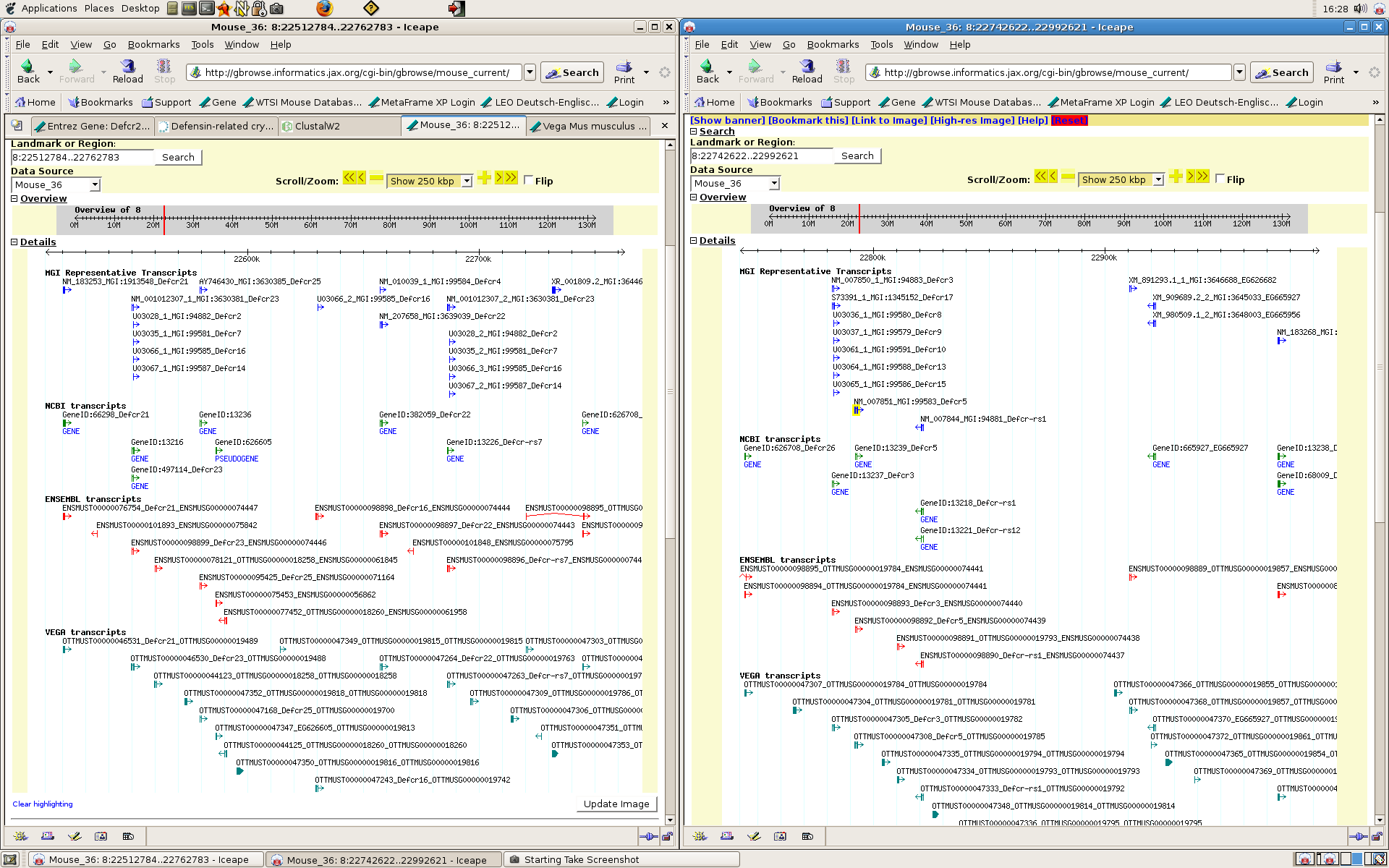


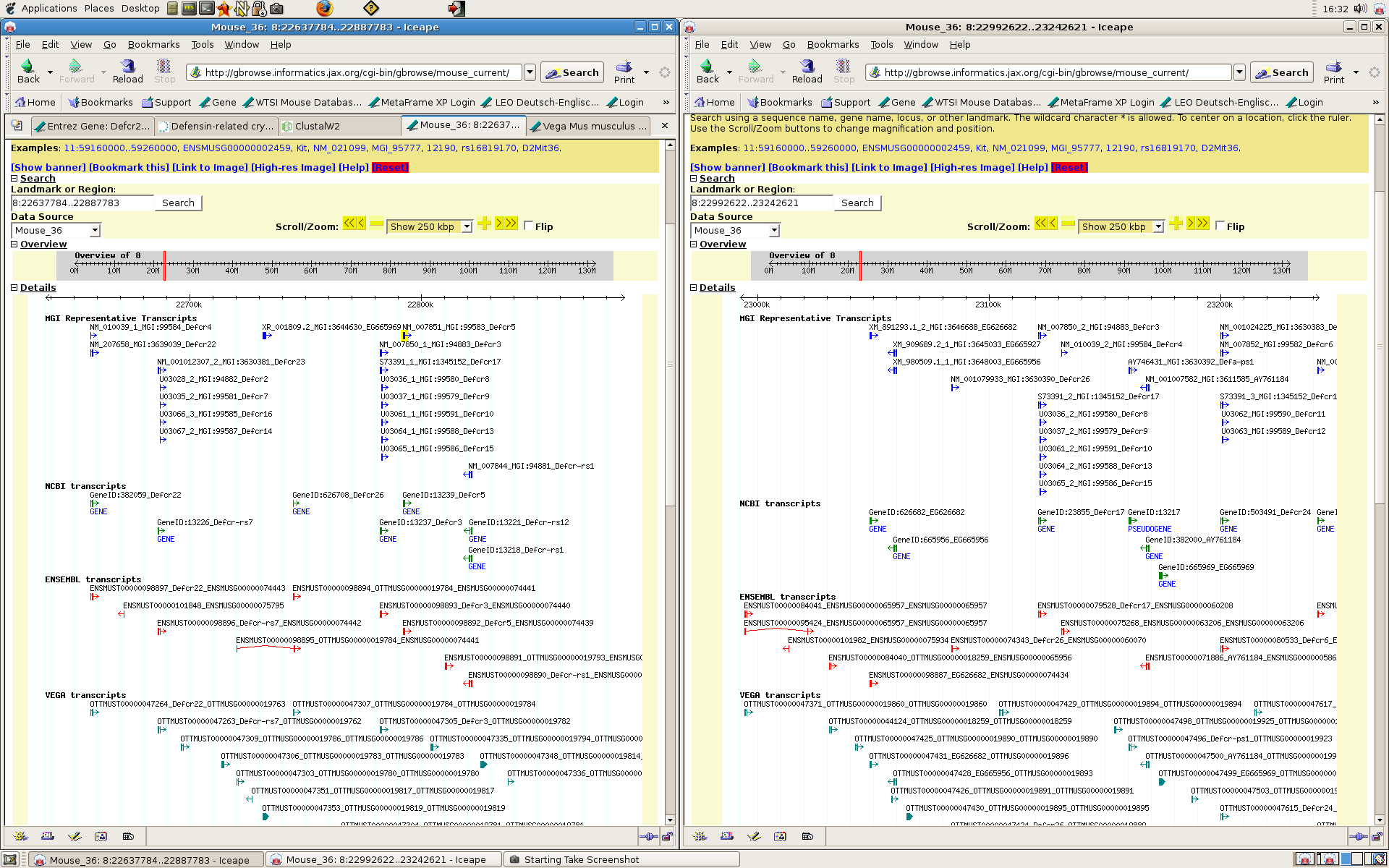
**Supplemental Figure S4:**

**Supplemental Figure S5: Mouse defensin gene cluster on chromosome8 in Ensembl based on NCBIM36**

**
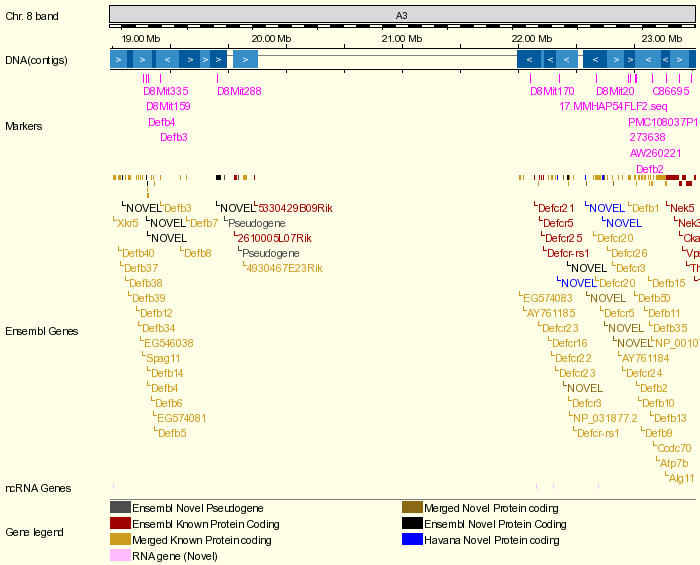
**

**Supplemental Figure S6: Human defensin gene cluster on chromosome 8 in Ensembl**

**
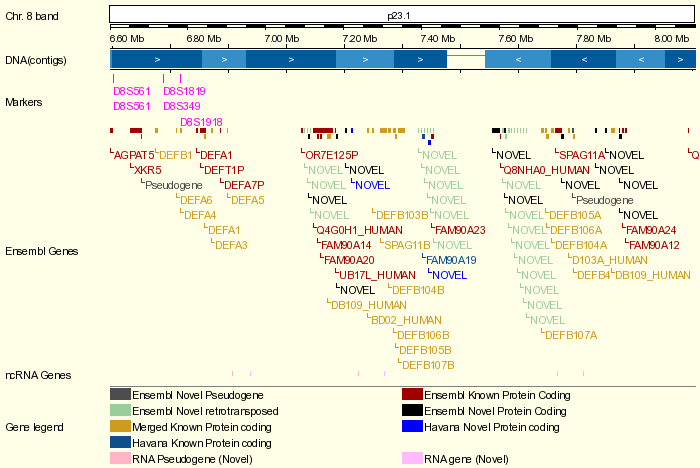
**

**Supplemental Figure S7: Rat defensin cluster on chromosome 16 (incomplete) in Ensembl**

**
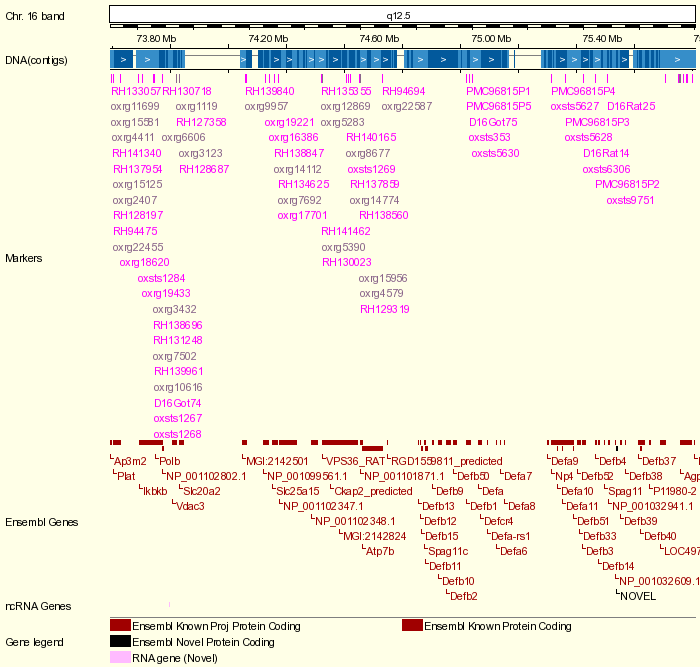
**
